# Demographically-Based Evaluation of Genomic Regions under Selection in Domestic Dogs

**DOI:** 10.1371/journal.pgen.1005851

**Published:** 2016-03-04

**Authors:** Adam H. Freedman, Rena M. Schweizer, Diego Ortega-Del Vecchyo, Eunjung Han, Brian W. Davis, Ilan Gronau, Pedro M. Silva, Marco Galaverni, Zhenxin Fan, Peter Marx, Belen Lorente-Galdos, Oscar Ramirez, Farhad Hormozdiari, Can Alkan, Carles Vilà, Kevin Squire, Eli Geffen, Josip Kusak, Adam R. Boyko, Heidi G. Parker, Clarence Lee, Vasisht Tadigotla, Adam Siepel, Carlos D. Bustamante, Timothy T. Harkins, Stanley F. Nelson, Tomas Marques-Bonet, Elaine A. Ostrander, Robert K. Wayne, John Novembre

**Affiliations:** 1 Department of Ecology and Evolutionary Biology, University of California, Los Angeles, Los Angeles, California, United States of America; 2 National Human Genome Research Institute, National Institutes of Health, Bethesda, Maryland, United States of America; 3 Department of Biological Statistics and Computational Biology, Cornell University, Ithaca, New York, United States of America; 4 CIBIO-UP, University of Porto, Vairão, Portugal; 5 ISPRA, Ozzano dell'Emilia, Italy; 6 Key Laboratory of Bioresources and Ecoenvironment, Sichuan University, Chengdu, China; 7 Department of Measurement and Information Systems, Budapest University of Technology and Economics, Budapest, Hungary; 8 ICREA at Institut de Biologia Evolutiva (CSIC-Universitat Pompeu Fabra), Barcelona, Spain; 9 Department of Computer Science, University of California, Los Angeles, Los Angeles, California, United States of America; 10 Bilkent University, Ankara, Turkey; 11 Estación Biológia de Doñana EBD-CSIC, Sevilla, Spain; 12 Department of Human Genetics, University of California, Los Angeles, Los Angeles, California, United States of America; 13 Department of Zoology, Tel Aviv University, Tel Aviv, Israel; 14 Department of Biology, University of Zagreb, Zagreb, Croatia; 15 Department of Biomedical Sciences, Cornell University, Ithaca, New York, United States of America; 16 Life Technologies, Foster City, California, United States of America; 17 Simons Center for Quantitative Biology, Cold Spring Harbor Laboratory, Cold Spring Harbor, New York, United States of America; 18 Stanford School of Medicine, Stanford, California, United States of America; 19 Centro Nacional de Analisis Genomico (CNAG/PCB), Baldiri Reixach 4–8, Barcelona, Spain; Uppsala University, SWEDEN

## Abstract

Controlling for background demographic effects is important for accurately identifying loci that have recently undergone positive selection. To date, the effects of demography have not yet been explicitly considered when identifying loci under selection during dog domestication. To investigate positive selection on the dog lineage early in the domestication, we examined patterns of polymorphism in six canid genomes that were previously used to infer a demographic model of dog domestication. Using an inferred demographic model, we computed false discovery rates (FDR) and identified 349 outlier regions consistent with positive selection at a low FDR. The signals in the top 100 regions were frequently centered on candidate genes related to brain function and behavior, including *LHFPL3*, *CADM2*, *GRIK3*, *SH3GL2*, *MBP*, *PDE7B*, *NTAN1*, and *GLRA1*. These regions contained significant enrichments in behavioral ontology categories. The 3^rd^ top hit, *CCRN4L*, plays a major role in lipid metabolism, that is supported by additional metabolism related candidates revealed in our scan, including *SCP2D1* and *PDXC1*. Comparing our method to an empirical outlier approach that does not directly account for demography, we found only modest overlaps between the two methods, with 60% of empirical outliers having no overlap with our demography-based outlier detection approach. Demography-aware approaches have lower-rates of false discovery. Our top candidates for selection, in addition to expanding the set of neurobehavioral candidate genes, include genes related to lipid metabolism, suggesting a dietary target of selection that was important during the period when proto-dogs hunted and fed alongside hunter-gatherers.

## Author Summary

Identification of the genomic regions under selection during dog domestication is extremely challenging because the demographic fluctuations associated with domestication can produce signals in polymorphism data that mimic those imposed by selective sweeps. We perform the first analysis of selection on the dog lineage that explicitly incorporates a demographic model, that by controlling for the rate of false discovery, more robustly identifies targets of selection. To do so, we conduct a selection scan using three wolf genomes representing the putative centers of dog domestication, two basal dog breeds (Basenji and Dingo), and a golden jackal as outgroup, for which we previously inferred a demographic model. We find that our demographically informed analyses filters out many signals that would be otherwise classified as putative selection signals under an empirical outlier approach. We identify 68 regions of the genome that have likely experienced positive selection. Besides identifying a number of new neurobehavioral candidate genes, our candidate regions contain genes related to lipid metabolism, including CCRN4L, which is centered in the 3^rd^ ranked region. This suggests a previously unreported locus of dietary adaptation, potentially due to the change in diet composition as hunting efficiency increased when proto dogs began hunting alongside hunter-gatherers.

## Introduction

Identifying regions of the genome that have undergone recent positive selection is central to understanding the causes of evolutionary diversification. Nevertheless, developing efficient and statistically robust methods for distinguishing genomic regions under selection from the neutral background expectation remains extremely challenging, particularly under complex, non-equilibrium demographic scenarios. The rapid rise in frequency of a new favorable allele typically leads to a reduced diversity in flanking regions as linked neutral polymorphism accompanies the adaptive substitution in a phenomenon known as genetic hitchhiking [1]. Many methods have been developed to detect such “selective sweep” signatures using genome-wide polymorphism data [2–4]. However, the distortions of the site-frequency spectrum (SFS) and/or extended linkage-disequilibrium accompanying episodes of positive selection can be difficult to distinguish from that produced by neutral processes related to a specific demographic history. For example, coalescent trees produced by population bottlenecks or founder events may be indistinguishable from those generated by selection [5,6], and in general, bottlenecks can generate long haplotypes that mimic those observed in selective sweeps [7]. Furthermore, population subdivision can produce counterintuitive and confounding effects [8,9]. Consequently, such demographic heterogeneity contributes to the low power and high false positive rates that can occur in genome-wide selection when using contemporary approaches [10–12].

The domestication of dogs from gray wolves is relevant to understanding the broader history of animal domestication and the genetic architecture of rapid phenotypic evolution [13,14]. As humans migrated out of Africa, they encountered gray wolves, which served as the founding stock for the domestic dog lineage. Archaeological remains [15–18], analyses of whole genome sequence data [19], and mitochondrial genomes of ancient and extant canid lineages [20] jointly support a pre-agricultural origin of dogs which was initiated by association with hunter-gatherers. During this initial interaction, selection for domestication traits was less intentionally directed by humans than it has been with the recent evolution of breed dogs, and instead, was predominantly an incidental by-product of human-wolf-prey interactions [21]. It is likely that dog domestication involved significant genetic changes in response to dietary and behavioral divergence from a wolf ancestor, and comparisons of brain-specific gene expression differences between dogs and wolves support the importance of the latter [22].

Identifying the targets of selection responsible for phenotypic divergence between wolves and dogs is hampered by the demographic complexity of the earliest phases of dog domestication, during which the ancestral dog lineage experienced at least one severe bottleneck and admixture with wolves occurred [19]. Such bottlenecks and admixture can bias selection scans that do not incorporate a demographic model, leading to false positive and negative signals, depending on the circumstances. Despite these potentially confounding effects, of the several studies investigating the genetic basis of phenotypic variation among recently formed breeds and early in domestication [23–28], none have formally modeled demography to generate a null, neutral expectation for patterns of variation.

Recently, we used coalescent-based analysis of whole genome sequence data from dogs and wolves to elucidate the complex demographic history underlying the domestication process. We estimated that domestication entailed a >16-fold reduction in effective population size (*N*_*e*_) for dogs, and a weaker, 3-fold reduction in wolves that began shortly after the initial dog-wolf divergence [19]. By comparison to modern wolves, earlier studies inferred a weaker domestication bottleneck [28–30], but the ancestral wolf bottleneck had not been previously known, and thus our results showed a greater loss of variation because dogs descended from a more variable ancestral wolf population. We also found evidence for considerable post-divergence admixture, not only between dogs and wolves, but also between wolves and golden jackals, and between golden jackals and the dog-wolf ancestor [19]. Recent admixture between dogs and wolves [31], and admixture between wolves and coyotes [32] had been previously detected, but the extent to which admixture events may obscure dog origins has only recently been appreciated [18,21,33,34]. This combination of bottlenecks and admixture substantially complicates efforts to distinguish between neutral processes and natural selection.

Previous investigations of selection on the dog lineage have taken an approach sometimes referred to as an empirical outlier scan for selection in which putatively selected regions are identified as outliers falling above some arbitrary value [13,25,27,35]. While this approach will detect loci under intense selection, controlling the rate of expected false positives is difficult because the distribution of test statistics under a null demographic model are not taken into account. Similarly, other recent studies of selection in domestic [36,37] and wild populations [38] have not accounted for demographic complexity. One difficulty is that a complete demographic model for large genome studies requires a time-consuming investigation of alternative scenarios that is computationally intensive.

To investigate positive selection on the dog lineage early in the domestication process and prior to the recent diversification of breeds, we re-examine patterns of polymorphism at 10 million single-nucleotide variant sites using six previously sequenced canid genomes that were used to infer a demographic model of dog domestication [19]. This sample included three wolves from Israel, Croatia, and China; two divergent dog breeds thought to be basal in the dog phylogeny, Basenji and Dingo; and a golden jackal [19]. Specifically, we use our previously inferred demographic model to calibrate a genome-wide scan for signatures of positive selection on the dog lineage and more confidently identify possible targets of recent positive selection while controlling for false positives. Although a recent genomic analysis of a wolf fossil has suggested a slower mutation rate for canids than used in our initial interpretation of our model [39], the raw parameter estimates from our model are independent of the mutation rate, i.e. our model explains the neutral distribution of polymorphism across our samples, regardless of the well-known uncertainty surrounding mutation rates. Finally, we contrast our findings with a demography-agnostic approach typical of previous studies. Our results expand the catalog of candidate neurobehavioral and dietary genes involved in domestication and provide candidates for future functional studies.

## Results

**Fig 1:**
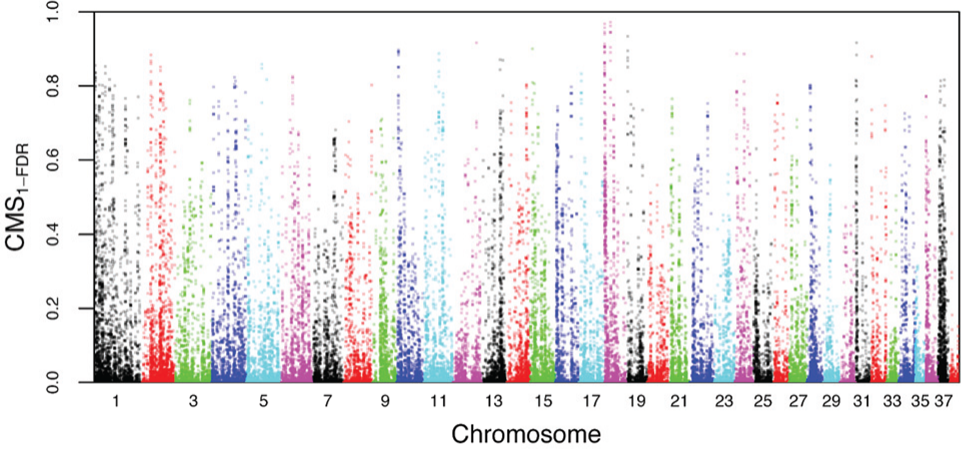
Distribution of CMS_1-FDR_ statistic calculated in 100kb sliding windows, with a 10kb step.

By leveraging the dataset of Freedman et al. [19], we were able to compute three summary statistics that are sensitive to the effects of positive selection in sliding windows across the dog reference genome [40]. These three statistics are as follows: 1) the difference in nucleotide diversity between dogs and wolves (Δπ); 2) F_ST_; and 3) the difference between dogs and wolves in Tajima’s D (Δ TD). After filtering on genome and sample level features, we computed summary statistics for 195,998 100kb sliding windows incremented in 10kb steps. Considerable variation was observed in the distribution of the three summary statistics (S1 Fig), and in our composite-of-multiple-signals statistic, comprised of the product of 1-FDR for those statistics (CMS_1-FDR_, see Methods and Materials; Fig 1). We used coalescent simulations based upon our previously constructed demographic model [19] to evaluate these signals relative to a genome-wide neutral expectation. Our model was inferred from a set of putatively neutral loci defined by a stringent set of filters with respect to features such as proximity to genic regions, degree of conservation, and the presence of segmental duplications. To estimate window-specific false discovery rates (q-values [41] from our empirical data), we conducted 200,000 simulations of 100kb windows with parameters fixed to the mean posterior values inferred for our demographic model (S12 Table in [19]). After calculating our three summary statistics for the simulated windows, for each statistic in each observed window, we calculated a p-value as the probability of observing in the simulated windows a value equal to or greater than that in the observed window. We then used the Benjamini-Hochberg procedure to calculate the probability of false discovery given that p-value as a means to correct for multiple comparisons [42].

To contrast the FDR-based findings with those not explicitly incorporating demography, we also identified outliers using an empirical outlier method. In this approach, we identified outlier regions as those comprised of the top 1% of all 100kb regions based on the joint percentiles of the underlying summary statistics (see Methods for details) which is similar to that used in a previous assessment of selection in dogs [35]. We collapsed windows into regions using the same criteria as in our FDR-based method.

### Regions under selection

**Fig 2:**
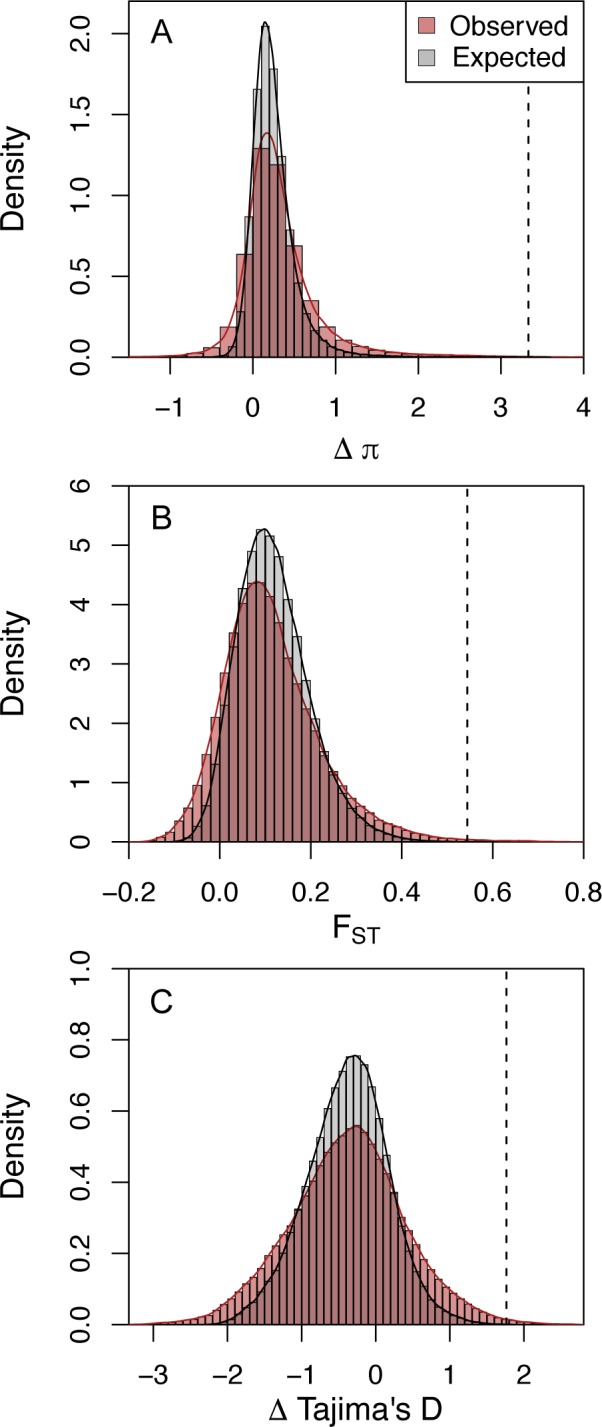
**Distributions of observed values for selection scan statistics and those computed from neutral coalescent simulations based up the inferred demographic history [19] for (A) Δπ, (B) F_ST_, and (C) Δ Tajima’s D.** Dashed lines indicate threshold values for FDR ≤ 0.01.

Comparison of our observed data to summary statistics observed in 200,000 simulations of 100kb windows under our previously inferred demographic model indicated a general over-dispersion of empirical windows relative to simulated ones. While some of this over-dispersion may be due to heterogeneity in genomic features (e.g. mutation rate) and the collective impact of various evolutionary processes, there is a clear excess of extreme values falling in the right-hand tails, outside the distribution of neutrally evolving windows, and consistent with the action of positive selection (Fig 2). Employing a false discovery rate (FDR) of 0.01 for Δπ, F_ST_, and Δ TD statistics, we identified 353, 827, and 982 windows, respectively, bearing signals consistent with positive selection (Table 1), for a total of 2081 unique windows. As an alternative approach, we repeated the procedure using null simulations with parameters drawn from the joint posterior distribution rather than fixing them at their mean posterior values (see Methods). The distributions of summary statistics were similar under both approaches (S2 Fig, Pearson correlations between FDR estimates between each approach >0.999, P < 2.2 × 10^−16^, 2558 unique windows identified). To be conservative, our subsequent analyses focus on the more limited set of 2081 windows found using both approaches.

**Table 1:** FDR threshold values and window counts for selection scan statistics.

Statistic	FDR	Windows <FDR	Minimum value ≤ FDR threshold	P at FDR
Δπ	0.05	519	3.127	1.25 × 10^−4^
	0.01	353	3.332	1.0 × 10^−5^
F_ST_	0.05	1495	0.481	3.8 × 10^−4^
	0.01	827	0.544	4.0 × 10^−5^
Δ Tajima’s D	0.05	2329	1.466	6.0 × 10^−4^
	0.01	982	1.763	5.0 × 10^−5^

After joining significant windows that were ≤ 200kb apart, both within and across statistics, 349 regions remained in total (S1 Table). These regions overlapped only partially with those identified in previous studies of selection in dogs. Specifically, 53 regions from previous studies were recovered using our approach, and additionally, we detected 296 novel regions.

With the 1% threshold, the empirical outlier approach identified 309 outlier regions. The overlap between the FDR-based and empirical outlier methods was low: 59% of the loci based on the FDR-based approach had no overlap with those from the empirical outlier method and 60% of empirical outliers had no overlap with the FDR-based approach (S3 Fig). Two patterns help to explain the low degree of overlap between the methods. First, looking at each summary statistic separately, the vast majority of windows in the top 1% of the empirical distribution have an FDR that would not pass our threshold of 0.01 (S4 Fig). A similar pattern is observed with the joint percentile statistics in that the vast majority of windows with an empirical joint percentile in the top 1% have high FDR for individual statistics (S5 Fig, red points), and in many cases more than one statistic. In both cases such outlier windows would be excluded using our FDR-based method. These results suggest that, in the absence of a baseline to assess if signals are consistent with neutral evolution, more than half of outliers in the empirical approach are not supported by an FDR-based approach, and many may actually be false positives. Furthermore, at the gene-level, the FDR and empirical outlier methods identify substantially different sets of genes, with 64% of genes identified in empirical outliers falling outside of FDR-identified regions. This suggests that inferences without demography might lead to mistaken functional interpretation of putative selection signals and gene ontology enrichments (S6 Fig).

**Fig 3:**
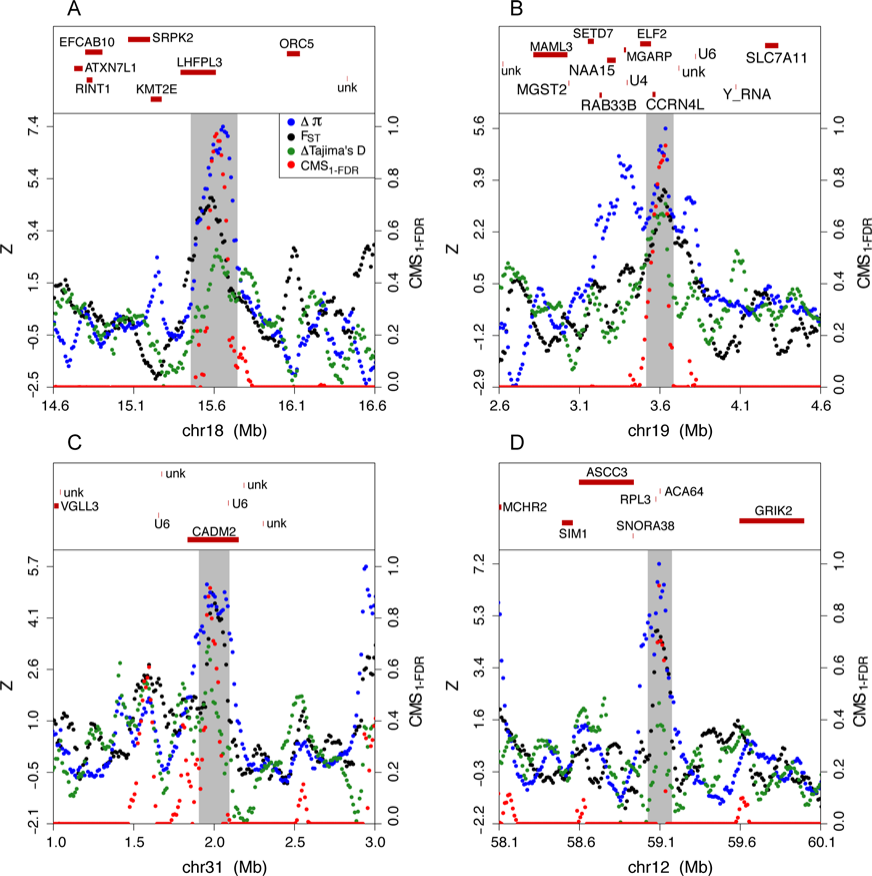
Z-transformed selection scan statistics, CMS_1-FDR_, and gene annotations within the (A) top ranked, (B) 3^rd^ ranked, (C) 4^th^ ranked, and (D) 5^th^ ranked candidate regions for positive selection on the dog lineage.

To rank the putative regions under selection we used a composite-of-multiple signals approach [43]. Specifically, we computed window-specific probabilities of false discovery (i.e. a false inference of deviation from neutrality) for our three summary statistics, and then computed the product of 1-FDR across those statistics to obtain a quantity we label CMS_1-FDR_. As the three summary statistics are not independent within windows, this product does not scale exactly with the weight of evidence for positive selection. Nevertheless, larger values of this statistic should indicate regions that are less likely to have been evolving neutrally. We used the maximum CMS_1-FDR_ statistic observed for any outlier window to rank windows and to localize the selection signal within each region (Fig 3). This statistic localizes the selection signal within outlier regions more tightly than computing a joint empirical percentile statistic (S7 Fig) which does not explicitly incorporate the probability of observing any of the constituent statistics under neutrality. When describing specific candidate genes likely under selection, we employ an additional filter in order to minimize false positives, by considering only genes within the top 100 regions.

**Fig 4:**
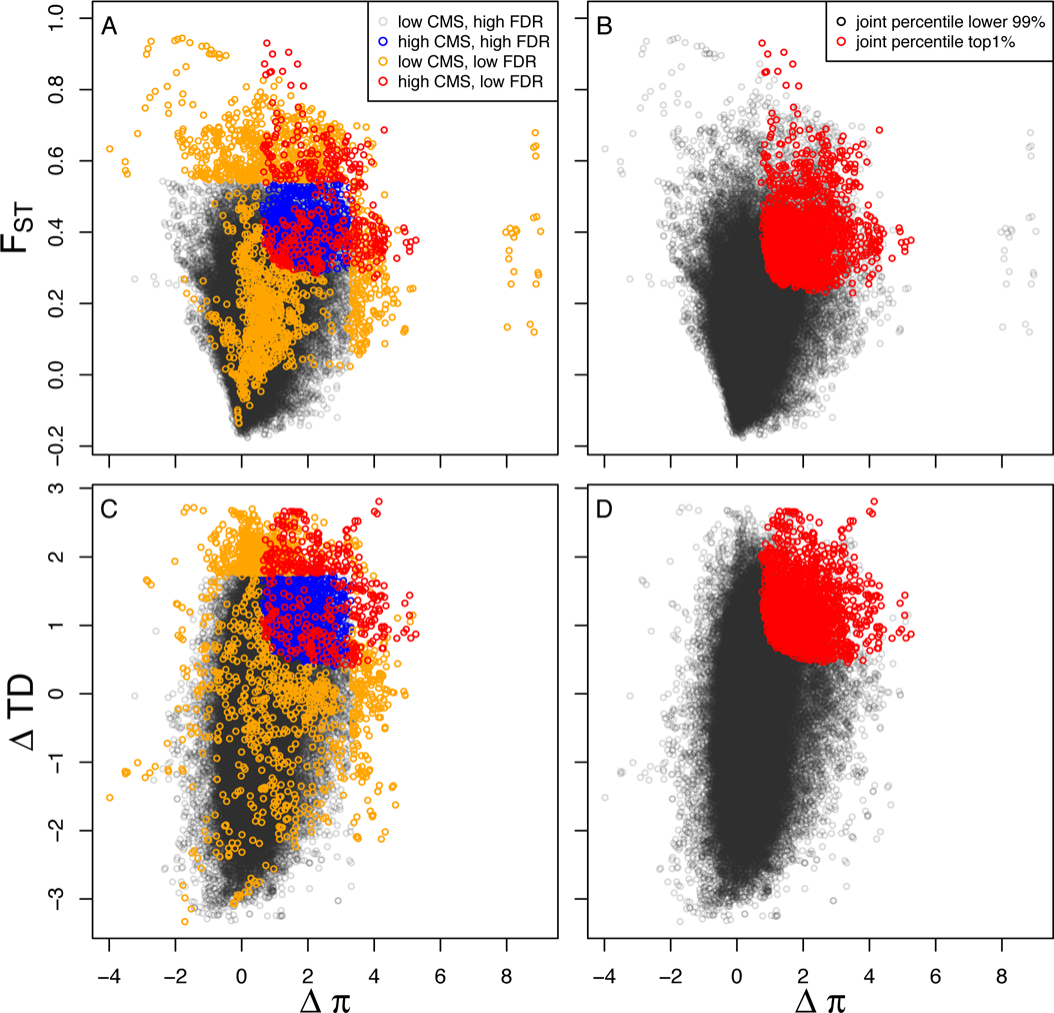
Biplots of summary statistics for 100kb sliding windows classified by their (A, C) CMS_1-FDR_ and (B, D) joint percentile. CMS_1-FDR_ is classified according to whether it is ≥ the minimum value observed in the top 100 regions for the maximum of CMS_1-FDR_ comprising the region (i.e. “high CMS”), and whether at least one summary statistic has an FDR ≤ 0.01 (i.e. low FDR). Thus, windows can be classified as “low CMS, high FDR”, “high CMS, high FDR”, “low CMS, low FDR”, and “high CMS, low FDR.” The first two categories are consistent with neutral expectations, the third is characterized by very weak evidence for selection, and the last category includes those windows with the strongest evidence for selection. For more details on these categories, see **Regions under selection** in Results.

The joint distribution of summary statistics, joint percentile, and CMS_1-FDR_ for 100kb window highlights the potential problems of not explicitly incorporating demography into selection scan for our set of genomes. To visualize these problems, we classify 100kb windows into four categories. The first category consists of those windows with both a low CMS_1-FDR_ statistic and high FDR for all three summary statistics, falling completely within neutral expectations (“low CMS, high FDR” in Fig 4A and 4C). It is possible for a window to have FDR≥0.01 for all three statistics, but still have low enough FDR such that CMS_1-FDR_ is comparable to that observed in outlier regions. We distinguish high CMS_1-FDR_ windows as those with a value for this statistic greater or equal to that observed in the top 100 ranked regions (i.e. the minimum across those 100 regions of the maximum value observed within a region). Thus, the second category consists of sites with FDR≥0.01 across all three summary statistics, but CMS_1-FDR_ above this threshold (“high CMS, high FDR” in Fig 4A and 4C). In some cases, windows have FDR≤0.01 for at least one summary statistic but there is at least one statistic with high FDR, such that they are classified as deviating from neutrality while having relatively low CMS_1-FDR_, beneath the threshold defined above (“low CMS, low FDR in Fig 4A and 4C). Finally, there are windows that have consistently low FDR across statistics such that CMS_1-FDR_ is high, owing to consistent signals of selection across statistics (“high CMS, low FDR in Fig 4A and 4C).

Based upon this classification of windows, we can distinguish different types of evidence for positive selection (Fig 4A and 4C). In contrast, many of the windows identified by the joint percentile method have high FDR for all three statistics (contrast blue points in Fig 4A and 4C with red points in Fig 4B and 4D), or have high enough FDR for some statistics such that CMS_1-FDR_ is low (contrast orange points in Fig 4A and 4C with red points in Fig 4B and 4D). However, by restricting our analysis to the top 100 windows we exclude regions that would be flagged by such low CMS_1-FDR_ windows that have very low support across all three summary statistics.

**Fig 5:**
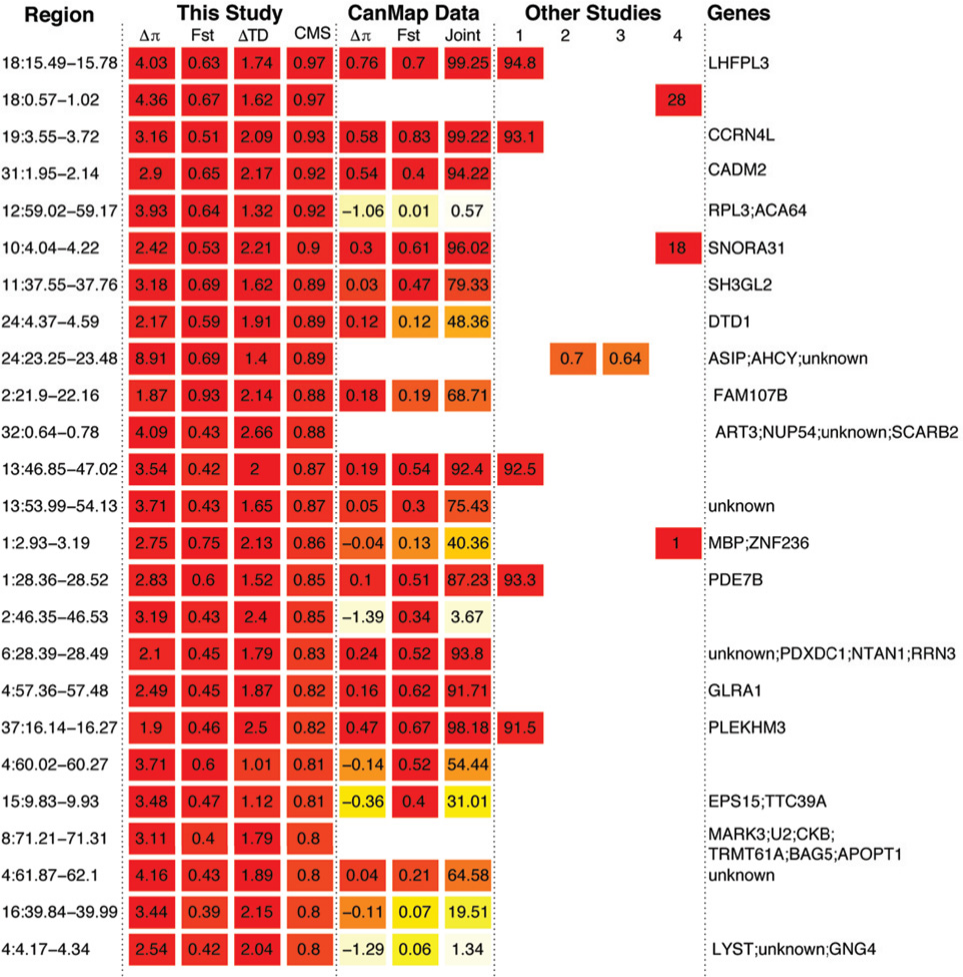
Top 25 outlier regions identified using the FDR-based methodology using Δout F_ST_, Δ Tajima’s D and validated with the 12-breed dog diversity panel (see text), with regions ranked according their respective maximum CMS_1-FDR_ statistic. Columns within “This study” are based on the sequencing data generated here, while those under “CanMap” are computed from a ~48k SNP data set for a large set of wolves and ancient/basal dog breeds [35]. Heat map colors reflect upper percentiles of the calculated metrics, with warmer colors indicating higher percentiles. Overlaps with previous studies: 1, vonHoldt et al. 2010 [35]; 2, Vaysse et al. (2011) [25]; 3, Boyko et al. (2010) [23]; and Axelsson et al. (2013), [27]; with numbers indicating the joint percentile, F_ST_, F_ST_ and region id, respectively for each study.

Key functional changes that derive from selection during the domestication process involve brain function and behavior [27,28,35], diet and metabolism [27], and pigmentation [44]. Consequently, we focus our discussion of the results on genes in regions showing evidence of a selective sweep with the FDR-based approach that are potentially relevant to these phenotypes. We only report genes that either overlap with the peak of the CMS_1-FDR_ statistic within an outlier region, or those that appear most proximate to that peak signal. As a further filter, we evaluated diversity patterns in 500kb intervals surrounding our top 100 outlier regions in a broader panel of 12 diverse breed dogs sequenced to approximately 40x mean coverage (SRA PRJNA288568). These sequence data include the dingo and basenji used in Freedman et al. [19] and genotypes were called for these data in a manner analogous to [19]. Based on these data, we excluded from further consideration any of the top 100 outlier regions where diversity in the 12-breed panel was greater or equal to that in adjacent non-outlier regions, or where the outlier region was centered on a localized reduction in diversity comparable to those seen in adjacent non-outlier intervals. This confirming data resulted in a reduced set of 68 regions. The filtered set of regions overlapped with only 21 previously identified candidate regions, and contained 47 novel regions (Fig 5 and S8 Fig).

In some cases, for any given outlier region, more than one gene may meet our criteria outlined above, such that highlighting particular genes will be *ad hoc*. Furthermore, it is possible that focusing on particular genes may exclude un-annotated regulatory elements that alter expression of downstream genes more distant from the statistical signal of selection. These caveats aside, we emphasize that our goal is to provide an updated list of candidate genes that can be used as a resource on which to base future investigations and functional assays, rather than to make absolute claims about the importance of any one gene to the domestication process. On a region-by-region basis, we document the extent to which the reported gene is the only one in the putative sweep region or whether it is the gene closest to the peak of the CMS_1-FDR_ statistic. Fig 5 and S8 Fig, provide a summary of the top regions we present given these considerations.

### Brain function/behavior genes

Eight of the top 20 candidate regions contain genes that have been implicated in neurological functions in other mammalian species. Our top region is centered on *LHFPL3*, a member of the lipoma *HMGIC* fusion partner family (Fig 3A). Mutations in *LHFPL3* have been detected in malignant glioma patients [45] and associated with autism risk [46]. *CADM2* is located within the 4th most extreme outlier region (Fig 3D) and is a synaptic cell adhesion molecule whose flanking regions show reduced homozygosity in autism patients [47]. *GRIK3* is the only gene within the 6^th^ region, and overlaps with the peak in the CMS_1-FDR_ signal. It is a glutamate receptor that has been associated with personality traits such as harm avoidance [48], schizophrenia and bipolar disorder [49], and was a neurobehavioral candidate gene in a selection scan of domestic cattle [36]. One cautionary note is that within this region our filters exclude large regions immediately adjacent to it, which raises the possibility that local genomic features might influence the quality of genotype calls.

*SH3GL2* is the only gene proximate to the peak in the CMS_1-FDR_ within the 8th ranked region and affects synaptic vesicle formation [50]. The peak signal in the 16^th^ ranked region is closest to *MBP*, a major constituent of the myelin sheath of oligodendrocytes and Schwann cells, and shown to be involved in schizophrenia [51]. *PDE7B*, which is the only gene overlapping the 17^th^ ranked region, is highly expressed in the brain and is involved in striatal functions related to dopaminergic pathways [52]. Inactivation of *NTAN1* (19^th^ region) in mice impairs spatial memory and leads to compensatory gains in non-spatial learning [53,54]. However, a RNA polymerase I-specific transcription initiation factor (*RRN3*) and *PDXDC1*, a gene with carboxylase activity associated with diverse phenotypes including renal carcinoma [55] and sensorineural hearing loss [56] were also either proximate to or overlapping the peak in CMS_1-FDR_ signal. *GLRA1* (the only gene in the 20^th^ region) mediates postsynaptic inhibition in the central nervous system, and mutations have been associated with startle disease [57]. For information on the remaining candidate genes with potential connections to behavior see S1 Text.

### Diet/lipid metabolism genes

In our 3^rd^ top outlier, the putative selection signature is most strongly peaked on *CCRN4L* (Fig 3B). *CCRN4L* (also known as Nocturnin) is expressed in a circadian fashion and studies in mice indicate that *CCRN4L* activates *PPAR*-γ, a gene that promotes bone adipogensis as opposed to osteoblast formation and that harbors a known diabetes risk variant in humans [58]. It also is known to regulate the expression of genes involved in lipogenesis and fatty acid binding, and knock-out mice are remarkable in being resistant to diet-induced obesity [58–61]. *CCRN4L* also suppresses *IGF1*, a well-known activator of bone growth [61] that underlies size variation amongst dog breeds [62,63]. The direction of these pleiotropic effects of *CCR4NL* implies a gain-of-function mutation would promote adipocyte formation, alter lipid metabolism, and suppress bone-growth.

Within our 9^th^ region, a second peak in CMS_1-FDR_ is centered on *SCP2D1*, a paralog of sterol carrier protein 2 (*SCP2*), which is highly expressed in genes involved in lipid metabolism, thought to function as an intracellular lipid transfer protein, and for which mice knockouts present altered lipid metabolism [64]. *PDXC1*, found within the 19^th^ region in addition to *NTAN1* (see above), is associated with plasma phospholipid concentrations and is functionally connected to the glycerophospholipid and sphingolipid pathways [65]. For information on additional candidate genes see S1 Text.

### Pigmentation candidate genes

The 10^th^ top region was centered on agouti signaling protein (ASIP), a well-known gene influencing pigmentation in mammals [66,67], that has a lesser known role in inhibiting lipolysis [68]. More recently, evidence is emerging that variation at *ASIP* can influence social behavior, most likely through its antagonistic effects on melanocortin receptors or α-melanocortin stimulating hormone [69,70]. Other than a small, predicted gene of unknown function, *LYST* is the only gene in the 30^th^ region. *LYST* not only overlaps the peak CMS_1-FDR_ signal, it overlaps the majority of the region as well. *LYST* has been associated with eye color variation in humans [71], and mutations can produce lighter skin and hair pigmentation [72].

### Characterizing dog-specific mutations in outliers

We found 8883 sites (2226 in outlier regions) containing dog-specific mutations that were at high allele frequency in the 12-breed panel (S9 Fig). Sites fixed between the dog and wolves we sequenced were enriched in outliers with respect to functional class relative to other genomic regions (χ^2^ = 23.06, df = 9, P = 6.1 × 10^−3^). The relative abundance of fixed differences in regions within one kb upstream of the transcriptional start site was twice that of the neutral background. Even so, there were only 12 upstream dog-specific mutations in outlier regions (S9 Fig), representing only 0.5% of all fixed sites in outlier regions. In contrast, the majority of dog-fixed sites fall within introns (29.2%) and putative intergenic (68.0%) regions. Only eight non-synonymous fixed sites were observed in outlier regions, and only five within regions that showed reduced diversity in the12-breed panel. Ensembl’s Variant Effect Predictor tool predicted that, for the transcript annotation displaying the maximum effect, all five variants were mutations of moderate effect. Associated SIFT predictions were as follows: SLK, in 115^th^ ranked region, low-confidence deleterious; two mutations in ACSBG2, 135^th^ ranked region, deleterious and tolerated, respectively; NOL8 (uncharacterized protein), 292.5^th^ ranked region, tolerated; ZNF585B, 292.5^th^ ranked region, tolerated. The one high confidence deleterious prediction based upon SIFT is in ACSBG2, which encodes a protein that is testis and brain-specific, and may play a role in spermatogenesis [73]. Nevertheless, the low frequency of dog-specific non-synonymous fixed sites and their occurrence within relatively low ranked outlier windows suggest coding mutations have been less important in the phenotypic divergence between dogs and wolves.

### Enrichment analyses

For enrichment analyses, we focused on the top 100 regions ranked by CMS_1-FDR_, minus those that did not also show reduced diversity in the 12-breed data set. We further filtered the gene set by only considering all genes that fell within 25kb of the peak in CMS_1-FDR_ within those regions. Based upon our requirement that FDR was ≤ 10%, we identified three categories that showed evidence of enrichment in the outlier regions. Notably, we found enrichments for behavior, locomotory behavior, and adult behavior (Table 2). However, after correction for multiple tests, none of these categories was significant. While it has been suggested that family-wise control of Type I errors is overly conservative for enrichment analyses [74], we consider our enrichment findings tentative, albeit consistent with the frequent appearance of brain function/behavior genes in our top hit regions.

**Table 2:** Enrichment categories discovered from the top 100 regions within 25kb of peak in joint statistic signal, excluding regions that fail to show reduced diversity in the 12-breed data set and categories with FDR >10%. Input and background total number of genes are 50 and 13,528, respectively.

Category	P	P-corrected^a^	FDR (%)	Background in Term	Fold Enrichment^b^	Genes
Biological process: behavior	0.0014	0.5877	2.1	469	4.6	DOCK2, GLRA1, LYST, ABAT, NTAN1, MBD2, ASIP, CXCL10
Biological process: locomotory behavior	0.0030	0.6163	4.4	274	5.9	DOCK2, GLRA1, LYST, ABAT, NTAN1, CXCL10
Biological process: adult behavior	0.0037	0.5421	5.4	86	12.6	GLRA1, ABAT, NTAN1, ASIP

^a^ Benjamini-corrected P-value.

^b^ (number of enriched genes in GO term/50)/(background genes in GO term/13528).

## Discussion

Extreme population bottlenecks are a hallmark of domestication events, and, in particular, demographic fluctuations and frequent admixture are regarded as important features of the evolutionary history of dogs [18,21,33]. We present the first effort to control for potential confounding effects of bottlenecks when inferring positive selection on regions of the dog genome, using a robust demographic model constructed from the same set of samples used to perform selection scans.

Two categories of genes continually emerged in the top half of our candidate regions list: those influencing behavior, neuropsychiatric disorders and brain function, and genes related to metabolism, in particular lipid metabolism. Genes associated with brain function and behavior are expected, given the dramatic shift from wild to domestic existence. However, genes related to fat metabolism are more surprising, and complement previous evidence for dietary adaptation occurring during domestication, particularly for increased starch metabolism [27]. Our 3^rd^ ranked region is nocturnin (*CCRN4L*). Evolution at this locus and at other metabolism genes (e.g. *ADRB2*, *DIP2C*, *PLCXD3*) may have facilitated shifts in lipid content of early domestic dog diets as they scavenged more on carcasses left behind by early humans. In fact, as incipient dogs and early humans began hunting together, prey capture rates may have increased relative to wild wolves and with it, the amount of lipid consumed by the assisting protodogs [75,76]. Unique dietary selection pressure may have resulted both from the amount consumed and the shifting composition of tissues that were available to protodogs after humans removed the most desirable parts of the carcass.

In addition to genes that may influence behavior and lipid metabolism, our selection scan also identified regions containing genes known to influence pigmentation. The effects of domestication on pigmentation are likely complex, potentially involving a combination of relaxed selection for crypsis, as well as positive selection for particular coat patterns [21]. The classic experiment selecting for tameness in foxes produced piebald and spotted coat color patterns after only 10 generations [44], suggesting that selection on pigmentation might not be direct but a by-product of selection on behavioral traits. While one investigation found no genetic correlation between coat coloration and tameness in rats [77], it is possible that in other species these two traits might be functionally coupled. As some of the pigmentation genes in our selection scans influence additional traits, the selection signals we detected may be produced by direct selection on early dog pigmentation phenotypes—the nature of which is not yet clear—or via other traits influenced by such putative pigmentation genes (e.g. the K locus, Anderson et al. [31]).

The general trend of reduced fear/aggression in domesticated species raises the possibility that this behavioral shift may have involved selection on the same set of genes in different domesticated species [78]. Although a comprehensive analysis of neurobehavioral candidates across species is beyond the scope of this paper, there are some notably parallelisms. In cats, both glutamate receptor (*GRIA1*) and protocadherin (*PCDHA1*, *PCDH4B*) genes show evidence of positive selection [79]. Similarly, in our top 10 candidate regions, we observe a glutamate receptor (*GRIK3*) which was also identified in a selection scan in cattle [36]. In contrast, top neurobehavioral candidate genes in rats did not overlap with our candidate gene set [80]. These comparisons suggest that positive selection during domestication may act on particular pathways, such as glutamate receptors, but not necessarily the same genes within those pathways.

The simultaneous appearance of multiple traits during domestication, labeled the “domestication syndrome,” raises fundamental questions concerning the genetic architecture of trait correlations. A recent, as of yet untested, hypothesis is that such correlations are functionally connected early in development during the processes of stem cell proliferation, differentiation, and migration [81]. Additionally, our results also suggest that pleiotropy may play a role in generating trait complexes observed in domestication species. For example, *CCRN4L* (our 3^rd^ top hit) directly influences lipid metabolism, but may indirectly reduce body size through suppressive effects on the well-established growth regulator *IGF1*. As an additional example, variation at agouti can influence lipid metabolism and behavior as well as pigmentation. While these examples may point to a mechanism facilitating the domestication syndrome, validation of potential pleiotropic effects among the candidate genes within our outlier regions will require analyses of tissue-specific expression and focused functional studies.

Our candidate regions contain a number of potential targets of selection not observed in recent selection scans, and only overlap to a small degree with regions detected by previous studies on dogs (Fig 5 and S8 Fig). While the lack of reproducibility of candidate regions among studies has raised questions concerning their general utility [82], we attribute discordance with prior studies to several factors. First, we employed a two-level filtering scheme on genotypes that included excluding genotypes intersecting with genome-level features such as copy number variants, where incorrect read mappings will distort allele frequency estimates and summary statistics that rely on those estimates. For example, the filters we used exclude the copy number variable amylase gene that had been reported previously as a crucial target of selection during dog domestication [27]. In that regard, one caveat of our study is that we only will detect adaptations based on copy number variants or structural variation through their effects on linked single nucleotide variation.

Second, methods that explicitly incorporate demographic information will likely produce different results from those that do not. This is perhaps most clearly demonstrated by the lack of overlaps between our FDR-based and demography-free joint percentile method (Fig 5), the latter being characteristic of “empirical” approaches which can potentially miss key targets of selection and falsely identify others [82].

Third, the set of genomes evaluated can have an effect on which regions are identified. From our previous demographic analysis [19], we determined that admixture between dogs and wolves is geographically structured such that the probability of gene flow is higher for wolf and dog lineages that are geographically proximate. Thus, biased sampling of dogs towards particular breeds may confound selection scan results in dogs by revealing specific features of regional dog breeds and wolves. Interestingly, our candidate regions did show some overlaps with an empirical outlier approach using SNP chip genotyping data when we restricted our sampling to so called “ancient” breeds (Fig 5 and S8 Fig). This overlap presumably occurs because our genomes and the ancient breed panel both retain patterns of polymorphism typical of the earliest dogs.

Our model-based assessment of FDR represents the first effort to account for demography in understanding positive selection during dog domestication. While this should reduce false positives among our candidate regions, a few caveats are necessary. First, our analysis is based on a small number of genomes. As a result, our approach should provide sufficient power for sweeps that have been strong, but partial sweeps that have led to less dramatic changes in dog allele frequencies will likely be missed. The challenge for future work is to expand the number of genome sequences analyzed while grounding selection scans with a demographic model that considers the intricacies of population dynamics and inter-lineage admixture. In particular, modeling demography for dozens to hundreds of lineages will pose a substantial inferential and computational challenge. A second caveat is that partial sweeps and soft sweeps may be difficult to detect with the summary statistics used here. It has been recently suggested that soft sweeps are the dominant mode of adaptation in wild populations [83] but there is still considerable uncertainty [84]. A third caveat is that, despite employing a stringent set of filters on both genome and sample level features, we cannot rule out the possibility that clusters of genotyping errors may have occurred in samples from either the dog or wolf lineage, such that some outlier regions may be false positives. Finally, while our use of overlapping, sliding windows allows us to localize the peak signal in outlier regions, it does raise the issue of non-independence and how it affects our approach to controlling for false discovery rate. Previous work indicates that the Benjamini-Hochberg FDR correction should be robust to certain kinds of dependence structure if tests meet the “positive regression dependency on each from a subset” (PRDS) criterion [85]. Furthermore, evidence has been presented that linkage mapping and associated tests fulfill PRDS [86], and the dependency among statistics at SNPs in such cases should very similar to that observed among statistics computed over windows across the genome. Nevertheless, should some features of our genome scans violate PRDS, it would mean that our estimates of FDR would be slightly less conservative (although certainly more so than empirical outlier approaches). We consider this an area worthy of future investigation.

Regardless of which mode is dominant, future work will likely uncover additional loci that have undergone positive selection in canids. In particular, future analyses using a larger set of dog and wolf genomes should provide power for assessing potential changes in adaptive substitutions occurring in multiple canine lineages, particularly if a neutral expectation can be calculated using a demographic model inferred for this larger sample. Despite these concerns, our model-based approach identifies a substantial number of new behavioral, metabolic, and pigmentation candidate genes that may contribute to the remarkable success of the oldest domesticated species and the only large carnivore adapted to life with humans.

## Methods

### Genome sequencing, sequence alignment and genotyping

All sequence alignment, genotyping, and quality-filtering methods were described previously [19]. Genotypes for all six canid genomes in that study were benchmarked against high quality genotypes from the Illumina CanineHD BeadChip, and showed a high degree of concordance with the chip data (e.g., 99.4% − 99.9% of heterozygous genotypes are confirmed by the CanineHD BeadChip). Sequence data are available at http://www.ncbi.nlm.nih.gov/bioproject/PRJNA274504. Vcf files can be obtained via the Dryad data repository at doi:10.5061/dryad.sk3p7.

We chose lineages to sequence with the goal of elucidating the timing, demographic context, and geographic origins of dogs. We selected the Basenji and Dingo for sequencing, as they represent two divergent breeds basal on the dog phylogeny [35]. We also utilized the Boxer reference genome as an additional haploid chromosome set. The Chinese, Croatian, and Israeli wolves represent lineages sampled geographically from the three regions from which dogs were previously hypothesized to have originated (East Asia, Europe, and Middle East, respectively). The golden jackal was chosen as an outgroup. This sampling strategy is also informative for understanding selection early in the dog lineage, as it captures the range of variation found in both dogs and wolves, thus minimizing the confusion of selection signals from later, lineage-specific effects, such as might occur were we to bias sampling towards modern breeds of European origin. All genotypes initially generated in CanFam 3.0 reference genome coordinates by Freedman et al. [19] were converted to the most current version, CanFam 3.1.

### Mapping regions with recent selective sweeps

#### Summary statistics

Demographic factors, such as population expansions, bottlenecks, or population structure, are confounders that distorts expected signatures of recent positive selection [9,11,12,87]. Since most domesticated species experience a population bottleneck [88], and a frequent mode of selection during domestication is selection from standing variation [40,89], detection of positive selection during domestication is difficult. To detect selective sweeps on the dog lineage during domestication, we selected three statistics that have been shown to have the highest power to detect selection under these conditions [89]: F_ST_ [40], Δπ [40], and ΔTD [40,90]. We used a sliding window approach in which we divided the reference genome into overlapping windows of size 100kb with 10kb increments. For each 100kb-window, we computed summary statistics using only sites that passed the genome and sample-level filters [19]. We considered the boxer reference haplotype when we compute statistics within the dog sample or between the dog and wolf sample. Because our analysis included a mixture of haploid (boxer reference) and diploid samples, we calculated F_ST_ from estimates of nucleotide diversity, i.e. (π_between_ − π_within_)/ π_between_, where nucleotide diversities are average per site estimates calculated across all pass filter sites within a 100kb window. We computed Δπ as π_wolf_/π_dog_ in each window, and report values on a log-scale (i.e. log(Δπ)) and ΔTD is computed as the difference in Tajima’s D between the wolf and dog sequences. In cases where π_dog_ was zero, we added a small fractional increment so that Δπ would still be computable. In cases where no segregating sites within dogs exist in a window, we did not calculate ΔTD.

#### Window filtering

We obtained 195,998 sliding windows of size 100kb with 10kb increments genome-wide. We then discarded any windows in which the number of fully observed sites is less than 30kb, because it is more likely that those windows are within or close to repeat/CNV regions or regions of poor sequencing quality.

#### Identifying outlier regions

In order to minimize the confounding of neutral, demographic signals with those produced by positive selection, we developed an approach to control for the false positive rate with simulations. Using the posterior mean parameter estimates from the best-fitting model (Fig 5A in [19]), we first simulated 200,000 100 kb windows using the program ms [91] under the demographic model that we previously inferred from the same seven genomes (including boxer) analyzed here [19], and computed Δπ, F_ST_, and ΔTD for those windows. Next, for each window in our observed data, we computed the proportion of simulated windows with a test statistic ≥ that in an observed window, i.e. a p-value defined as the probability of the observed data under neutrality. We then computed FDR using the Benjamini-Hochberg procedure for our set of windows based upon our empirical data. For each test statistic, we retained windows with FDR≤0.01. We considered the effects of simulation strategy on FDR calculations and outlier window identification by drawing 1000 samples from the joint posterior distribution of our best-fitting demographic model and simulating each of these samples 200 times for a total of 200,000 100kb windows. As the sliding window approach means that windows are often clustered across the genome (Fig 1), we collapsed windows across statistics into outlier regions if they were within 200kb of each other. As a heuristic to rank outlier regions, we took a “composite of multiple signals” (CMS) approach, similar to that employed in a recent selection scan in humans [43]. Specifically, we computed CMS_1-FDR_ as (1-FDR_Δπ_)*(1-FDR_FST_)*(1-FDR_ΔTD_), and ranked regions according to the maximum CMS_1-FDR_ observed in any of the 100kb windows comprising a region. We note that given Δ Tajima’s D cannot be calculated for windows with no polymorphism, we have excluded windows with strong signals in F_ST_ and Δπ that are potentially of interest (orange points on far right of Fig 4A).

To contrast our demography-informed approach of controlling for FDR with neutral simulations, we examined overlap of outliers based upon this approach with a fundamentally different, “demography-free” approach that examines an arbitrary percentage of a top set of outliers, similar to many genome-wide selection scans that don’t explicitly include demography. In this approach, for each summary statistic (F_ST_, Δπ and ΔTD), we computed empirical percentiles by ranking each window by the summary statistic in question and transforming the ranks to percentiles (% F_ST_, % Δπ and % ΔTD). We then calculated a “joint” empirical percentile by computing the product of the empirical percentiles obtained for the three summary statistics in each window [(% Product) = (% F_ST_) * (% Δπ) * (% ΔTD)] and then ranking each window by the products (% Product) and transforming the ranks to percentiles (% Joint). In order to draw Manhattan plots, we transformed the joint empirical percentiles defined for each window into joint empirical p-values. Joint empirical p-values are defined as a probability of obtaining a joint empirical percentile greater than or equal to that observed for the window in question. For the joint empirical percentile, we defined the top 1% windows as outlier windows. As with our primary approach described above, windows ≤ 200kb apart were collapsed into outlier regions. For this method, we ranked outlier regions by the maximum joint percentile.

### Dog-specific mutations

For our analysis of the distribution of sites fixed between dogs and wolves, we first identified sites where the Basenji and Dingo were homozygous for the Boxer reference derived allele, and where the three wolves and golden jackal were fixed for an alternative (i.e. the ancestral) allele. We then reduced this set of candidate sites by only including sites where the dog derived allele was observed across the 12-breed genome sequences at a frequency ≥ 0.75. We evaluated the functional consequences of dog-specific non-synonymous variants using Ensembl’s Variant Effect Predictor (http://www.ensembl.org/info/docs/tools/vep/index.html).

### Enrichment analyses

To detect functional enrichment within the genes intersecting our outlier regions, we used the program DAVID [74], with the *Canis lupus* gene set as background. We focused on the genes falling within 25kb of the peak CMS_1-FDR_ signal for the top 100 regions, minus those regions that did not also show a reduction in diversity in the 12-breed data set. Because enrichment analyses require a relatively large input set of genes in order to detect enrichment patterns, and given that we already perform statistical inference to identify regions under selection, we report all categories with FDR ≤ 10%. We also report uncorrected P-values as well as P-values corrected for multiple comparisons using Benjamini’s method, although the latter are generally considered to be extremely conservative [74].

## Supporting Information

S1 Text Description of additional candidate genes. (PDF)

S1 Table Outlier regions identified with the FDR-based method, ranked according to CMS_1-FDR_. (XLS)

S1 Fig Genome-wide distribution of Δπ, F_ST_, Δ Tajima’s D in 100kb sliding windows. (PDF)

S2 Fig Comparison of distributions computed from neutral coalescent simulations based up the posterior mean parameter estimates from the inferred demographic history, [19] and 1000 samples from the joint posterior distribution for (A) Δπ, (B) F_ST_, and (C) Δ Tajima’s D.
(PDF)

S3 Fig Distribution of overlaps between outlier regions detected between methods for FDR and empirical outlier methods. (PDF)

S4 Fig Bi-plots of empirical percentile vs. FDR for individual summary statistics across 100 kb windows, demonstrating that the majority of windows in the top 1% have FDR > 0.01. (A) Entire range of empirical percentile and (B) Focus on the top 20% of the empirical distribution. Horizontal and vertical dotted lines indicate the 99^th^ percentile and 1% FDR, respectively.
(PDF)

S5 Fig FDR of individual statistics vs. the joint percentile statistic for 100kb windows, used to identify outlier windows in the empirical outlier (non-FDR) approach, for (A) Δπ, (B) F_ST_, and (C) Δ Tajima’s D.
(PNG)

S6 Fig Venn diagram displaying overlap of candidate gene sets obtained with FDR-based and empirical outlier (EO) methods for detecting positive selection on the dog lineage. Genes unique to empirical methods relative to FDR methods are those falling within windows with a high false discovery rate (and thus are likely to be enriched with false positives)
(PDF)

S7 Fig Distribution of CMS_1-FDR_ and the joint percentile statistic for the top and 3^rd^ ranked regions, demonstrating that CMS_1-FDR_ localizes the peak of the outlier region signal more precisely than the joint percentile. (PDF)

S8 Fig All 68 outlier regions identified using the FDR-based methodology using Δπ, F_ST_, Δ Tajima’s D that were validated with the 12-breed dog diversity panel. Columns within “This study” are based on the sequencing data generated here, while those under CanMap are computed from a ~48k SNP data set for a large set of wolves and ancient/basal dog breeds. Heat map colors reflect upper percentiles of the calculated metrics, with warmer colors indicating higher percentiles. Overlaps with previous studies: 1, vonHoldt et al. 2010 [35]; 2, Vaysse et al. (2011) [25]; 3, Boyko et al. (2010) [23]; and Axelsson et al.(2013), [27], with numbers indicating the joint percentile, F_ST_, F_ST_, and region id, respectively for each study.
(PDF)

S9 Fig Distribution of sites fixed between dogs and wolves in neutral and outlier regions according to functional class, filtered according to the requirement that the dog-specific allele be at a frequency of 0.75 or greater among a panel of 12 additional breed dogs. Numbers above bars indicate counts of fixed sites.
(PDF)
