# Supplementary material for: Demographically-Based Evaluation of Genomic Regions under Selection in Domestic Dogs
Source: PLoS Genet. 2016 Mar 4;12(3):e1005851. doi: 10.1371/journal.pgen.1005851 (PMC4778760; doi:10.1371/journal.pgen.1005851)
Supplement: S1 Text — (PDF) [file pgen.1005851.s001.pdf]

Demographically-Based Evaluation of Genomic Regions Under Selection in Domestic Dogs

Adam H. Freedman[1*], Rena M. Schweizer[2], Diego Ortega-Del Vecchyo[2], Eunjung Han[2], Brian Davis[3], Ilan Gronau[4], Pedro M. Silva[5], Marco Galaverni[6], Zhenxin Fan[7], Peter Marx[8], Belen Lorente-Galdos[9], Oscar Ramirez[9], Farhad Hormozdiari[10], Can Alkan[11], Carles Vilà[12], Kevin Squire[13], Eli Geffen[14], Josip Kusak[15], Adam R. Boyko[16], Heidi G. Parker[3], Clarence Lee[17], Vasisht Tadigotla[17], Adam Siepel[18], Carlos D. Bustamante[19], Timothy T. Harkins[17], Stanley F. Nelson[13], Tomas Marques-Bonet[9,20], Elaine A. Ostrander[3], Robert K. Wayne[2], John Novembre[21]

[1]Faculty of Arts and Sciences Informatics Group, Harvard University, Cambridge, MA.
[2]Department of Ecology and Evolutionary Biology, University of California, Los Angeles, Los Angeles, CA, USA.
[3]National Human Genome Research Institute, National Institutes of Health, Bethesda, MD, USA.
[4]Department of Biological Statistics and Computational Biology, Cornell University, Ithaca, NY, USA.
[5]CIBIO-UP, University of Porto, Vairão, Portugal.
[6]ISPRA, Ozzano dell'Emilia, Italy.
[7]Key Laboratory of Bioresources and Ecoenvironment, Sichuan University, Chengdu, China.
[8]Department of Measurement and Information Systems, Budapest University of Technology and Economics, Budapest, Hungary.
[9]ICREA at Institut de Biologia Evolutiva (CSIC-Universitat Pompeu Fabra), Barcelona, Spain.
[10]Department of Computer Science, University of California, Los Angeles, Los Angeles, CA, USA.
[11]Bilkent University, Ankara, Turkey.
[12]Estación Biológia de Doñana EBD-CSIC, Sevilla, Spain.
[13]Department of Human Genetics, University of California, Los Angeles, Los Angeles, CA, USA.
[14]Department of Zoology, Tel Aviv University, Tel Aviv, Israel.
[15]Department of Biology, University of Zagreb, Zagreb, Croatia.
[16]Department of Biomedical Sciences, Cornell University, Ithaca, NY.
[17]Life Technologies, Foster City, CA, USA.
[18]Simons Center for Quantitative Biology, Cold Spring Harbor Laboratory, Cold Spring Harbor, NY, USA.
[19]Stanford School of Medicine, Stanford, CA, USA.
[20]Centro Nacional de Analisis Genomico (CNAG/PCB). Baldiri Reixach 4-8, 08023 Barcelona.
[21]Department of Human Genetics, University of Chicago, Chicago, IL, USA.
*Correspondence to: adamfreedman@fas.harvard.edu

Description of additional candidate genes

Brain function/behavior

The 27th region overlaps or is proximate to several genes including MARK3 (see below); BAG5, which interacts with Parkinson's disease-related genes LRRK2 [1] and Parkin [2]; and CKB, which shows increased transcription in the brain during development and co-expression with MBP in rats [3] (but also appears to play an important role in bone resorbtion in osteoclasts [4]). CUX2 (the only gene overlapping the 37th region as well as the peak in $CMS_{1\text{-}FDR}$ signal) is a key marker of neuronal fate during mammalian cortex development [5] whose knockout in mice shows deficits in working memory [6]. Mutations in ARID1B (the only gene in the 39th region, also overlapping with the $CMS_{1\text{-}FDR}$ peak) cause developmental delays, and the absence of 5th finger and toenail, suggesting a connection to both behavior and formation of the dewclaw [7]. The peak of the $CMS_{1\text{-}FDR}$ signal in the 42nd region overlaps with LRRN3, and is an autism candidate gene [8]. The only other gene (IMMP2L) found within this large 430kb region is at the opposite end and far from the peak signal. TMEM132D is a large gene overlapping the 45th region in its entirety and is the sole gene that intersects this region. Mutations in TMEM132D are associated with anxiety phenotypes [9], and it was identified in a selection scan as domestication candidate gene in cattle [10].

Two g-coupled protein receptors lie at either end and just outside of the 53rd region, which does not intersect directly with any gene annotations. However, GPR139 is proximate to the peak in $CMS_{1\text{-}FDR}$ signal, and is associated with inattention/ADHD [11]. SEMA3D is the only gene within the 69th region, and spans its entire length. This gene is involved in peripheral axon guidance during development [12], and mutations within it may be associated with schizophrenia [13]. MBD2 is the only gene of three (including POLI and STARD6) that overlaps with the peak in $CMS_{1\text{-}FDR}$ in the 88th region. It activates glucocorticoid receptor, in doing so modulates patental care in rats, and hippocampal expression of MBD2 increases in response to maternal licking [14]. Although a small gene of unknown function is contained within the 98th region, it is spanned in its entirety by PARK2. PARK2 produce the protein parkin, and mutations in PARK2 are associated with Parkinson's disease (http://omim.org/entry/602544).

Diet/lipid metabolism

ADRB2 (23rd) is an andrenergic receptor that regulates energy balance, and belongs to a class of receptors that stimulates lipid mobilization in adipose tissue. Mutations in and downregulation of ADRB2 are associated with obesity [15,16]. Melanin concentrating hormone receptor 2 (MCHR2, 95th) plays a role in behavioral aspects of food intake, and energy homeostasis [17], and may act antagonistically to MCHR1 so as to reduce food intake and diet-induced obesity [18]. However, MCHR2 was also recently identified as a candidate gene in a GWAS on age of menarche onset [19] so which phenotype might have been acted on by selection remains unclear. RAPH1 (99th) has been associated with both mood disorders [20] and bloodstream concentrations of butyrylcholinesterase [21], the activity of which influences lipid concentrations levels and obesity.

Supplementary References

1. Beilina A, Rudenko IN, Kaganovich A, Civiero L, Chau H, et al. (2014) Unbiased screen for interactors of leucine-rich repeat kinase 2 supports a common pathway for sporadic and familial Parkinson disease. Proceedings of the National Academy of Sciences of the United States of America 111: 2626-2631.
2. Kalia SK, Lee S, Smith PD, Liu L, Crocker SJ, et al. (2004) BAG5 inhibits parkin and enhances dopaminergic neuron degeneration. Neuron 44: 931-945.
3. Shen W, Willis D, Zhang YP, Schlattner U, Wallimann T, et al. (2002) Expression of creatine kinase isoenzyme genes during postnatal development of rat brain cerebellum: evidence for transcriptional regulation. Biochemical Journal 367: 369-380.
4. Chang EJ, Ha J, Oerlemans F, Lee YJ, Lee SW, et al. (2008) Brain-type creatine kinase has a crucial role in osteoclast-mediated bone resorption. Nature Medicine 14: 966-972.
5. Franco SJ, Gil-Sanz C, Martinez-Garay I, Espinosa A, Harkins-Perry SR, et al. (2012) Fate-Restricted Neural Progenitors in the Mammalian Cerebral Cortex. Science 337: 746-749.
6. Cubelos B, Sebastian-Serrano A, Beccari L, Calcagnotto ME, Cisneros E, et al. (2010) Cux1 and Cux2 Regulate Dendritic Branching, Spine Morphology, and Synapses of the Upper Layer Neurons of the Cortex. Neuron 66: 523-535.
7. Santen GWE, Aten E, Sun Y, Almomani R, Gilissen C, et al. (2012) Mutations in SWI/SNF chromatin remodeling complex gene ARID1B cause Coffin-Siris syndrome. Nat Genet 44: 379-380.
8. Hutcheson HB, Olson LM, Bradford Y, Folstein SE, Santangelo SL, et al. (2004) Examination of NRCAM, LRRN3, KIAA0716, and LAMB1 as autism candidate genes. BMC Med Genet 5: 12.
9. Erhardt A, Czibere L, Roeske D, Lucae S, Unschuld PG, et al. (2011) TMEM132D, a new candidate for anxiety phenotypes: evidence from human and mouse studies. Molecular Psychiatry 16: 647-663.
10. Qanbari S, Pausch H, Jansen S, Somel M, Strom TM, et al. (2014) Classic Selective Sweeps Revealed by Massive Sequencing in Cattle. PLoS Genet 10.
11. Ebejer JL, Duffy DL, van der Werf J, Wright MJ, Montgomery G, et al. (2013) Genome-Wide Association Study of Inattention and Hyperactivity-Impulsivity Measured as Quantitative Traits. Twin Research and Human Genetics 16: 560-574.
12. Liu Y, Halloran MC (2005) Central and peripheral axon branches from one neuron are guided differentially by Semaphorin3D and transient axonal glycoprotein-1. J Neurosci 25: 10556-10563.
13. Fujii T, Uchiyama H, Yamamoto N, Hori H, Tatsumi M, et al. (2011) Possible association of the semaphorin 3D gene (SEMA3D) with schizophrenia. Journal of Psychiatric Research 45: 47-53.
14. Weaver ICG, Hellstrom IC, Brown SE, Andrews SD, Dymov S, et al. (2014) The methylated-DNA binding protein MBD2 enhances NGFI-A (egr-1)-mediated transcriptional activation of the glucocorticoid receptor. Philosophical Transactions of the Royal Society B-Biological Sciences 369.
15. Naka I, Hikami K, Nakayama K, Koga M, Nishida N, et al. (2013) A functional SNP upstream of the beta-2 adrenergic receptor gene (ADRB2) is associated with obesity in Oceanic populations. International Journal of Obesity 37: 1204-1210.

16. Jalba MS, Rhoads GG, Demissie K (2008) Association of codon 16 and codon 27 beta 2-adrenergic receptor gene polymorphisms with obesity: A meta-analysis. Obesity 16: 2096-2106.
17. Pissios P, Bradley RL, Maratos-Flier E (2006) Expanding the scales: The multiple roles of MCH in regulating energy balance and other biological functions. Endocrine Reviews 27: 606-620.
18. Chee MJS, Pissios P, Prasad D, Maratos-Flier E (2014) Expression of Melanin-Concentrating Hormone Receptor 2 Protects Against Diet-Induced Obesity in Male Mice. Endocrinology 155: 81-88.
19. Elks CE, Perry JRB, Sulem P, Chasman DI, Franceschini N, et al. (2010) Thirty new loci for age at menarche identified by a meta-analysis of genome-wide association studies. Nat Genet 42: 1077-U1073.
20. Redei EE, Andrus BM, Kwasny MJ, Seok J, Cai X, et al. (2014) Blood transcriptomic biomarkers in adult primary care patients with major depressive disorder undergoing cognitive behavioral therapy. Translational Psychiatry 4.
21. Benyamin B, Middelberg RP, Lind PA, Valle AM, Gordon S, et al. (2011) GWAS of butyrylcholinesterase activity identifies four novel loci, independent effects within BCHE and secondary associations with metabolic risk factors. Hum Mol Genet 20: 4504-4514.
