# Supplementary figures and images for: Demographically-Based Evaluation of Genomic Regions under Selection in Domestic Dogs
Source: PLoS Genet. 2016 Mar 4;12(3):e1005851. doi: 10.1371/journal.pgen.1005851 (PMC4778760; doi:10.1371/journal.pgen.1005851)

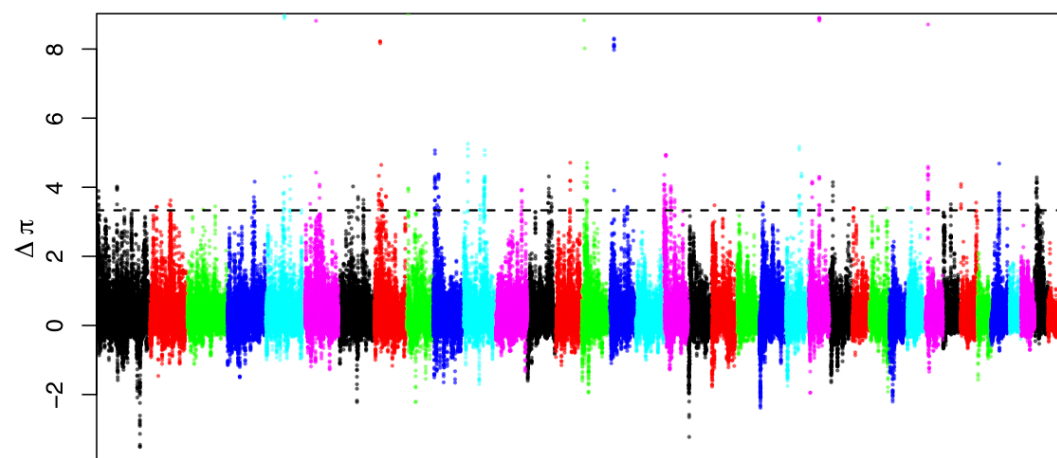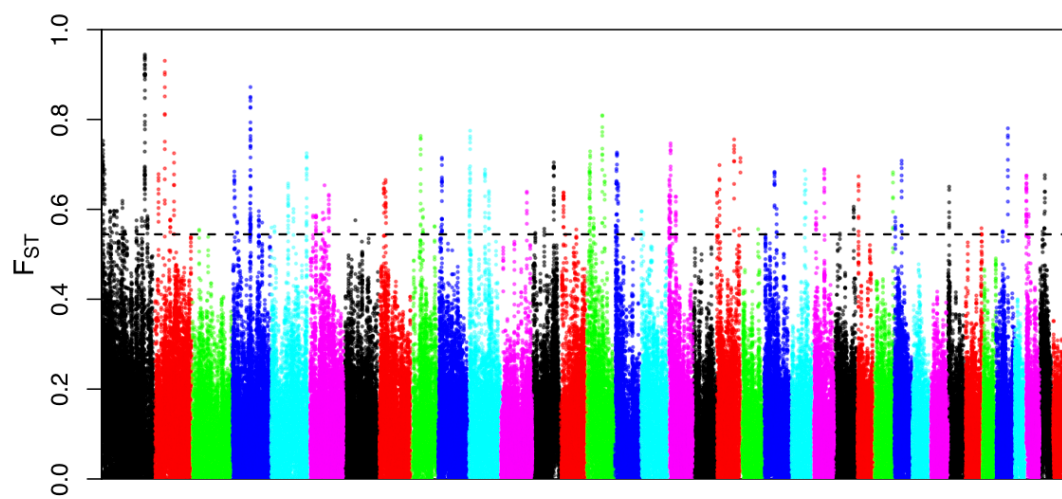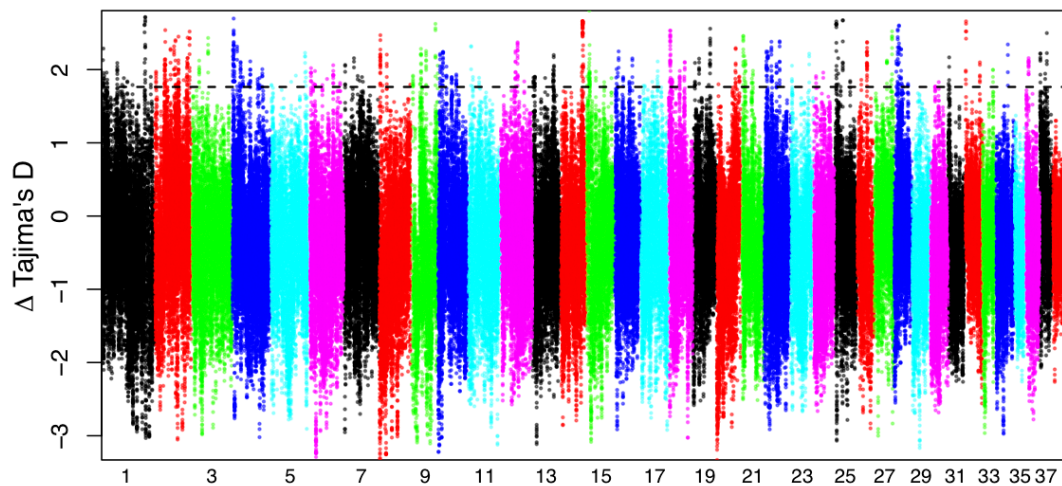

Supplement: S1 Fig — (PDF) [file pgen.1005851.s003.pdf]

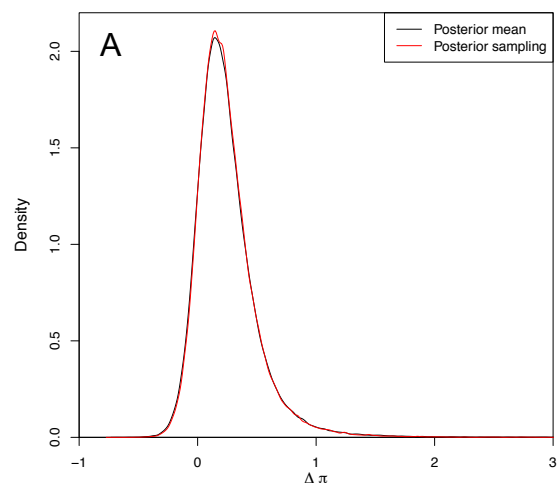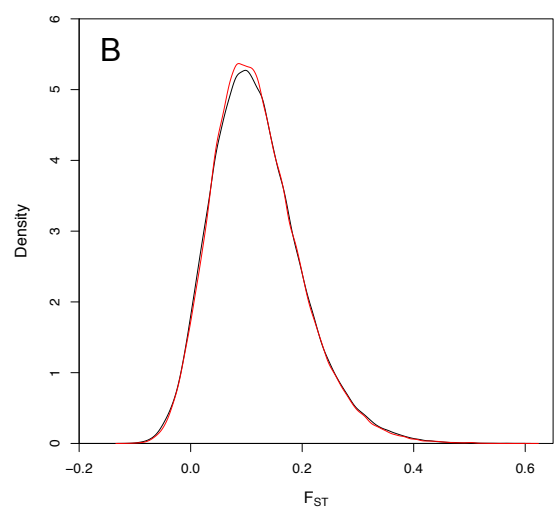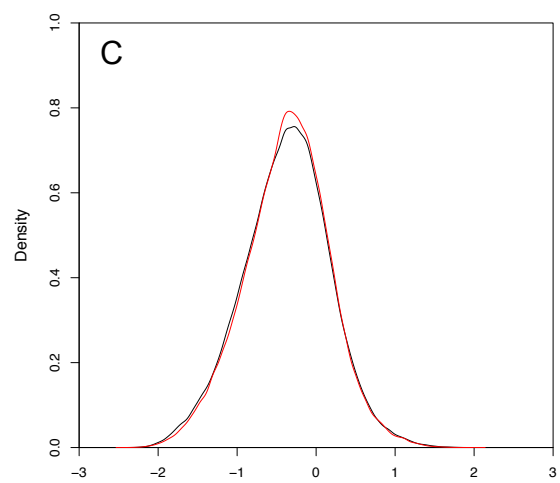

Supplement: S2 Fig — Comparison of distributions computed from neutral coalescent simulations based up the posterior mean parameter estimates from the inferred demographic history, [19] and 1000 samples from the joint posterior distribution for (A) Δπ, (B) FST, and (C) Δ Tajima’s D. (PDF) [file pgen.1005851.s004.pdf]

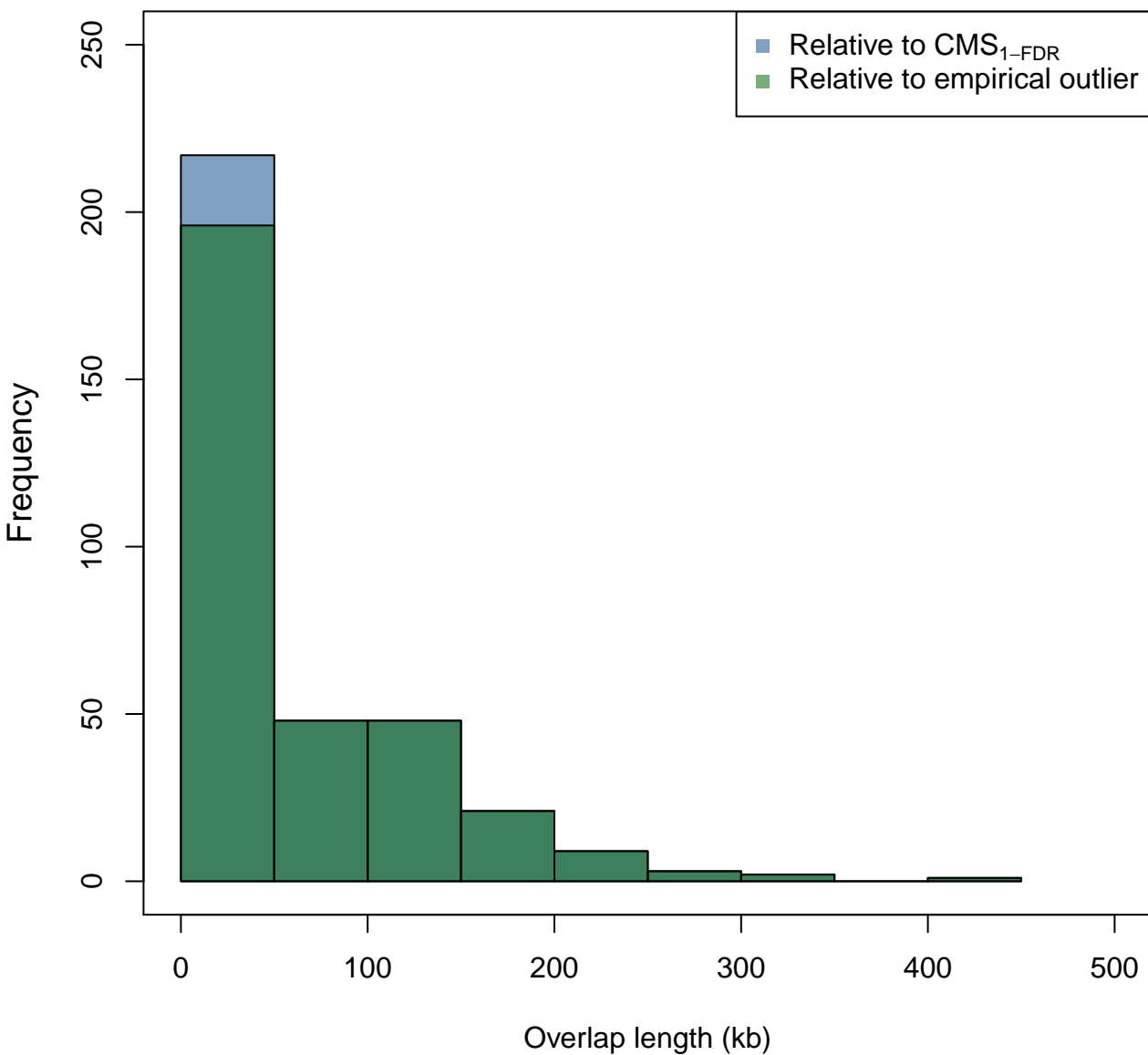

Supplement: S3 Fig — (PDF) [file pgen.1005851.s005.pdf]

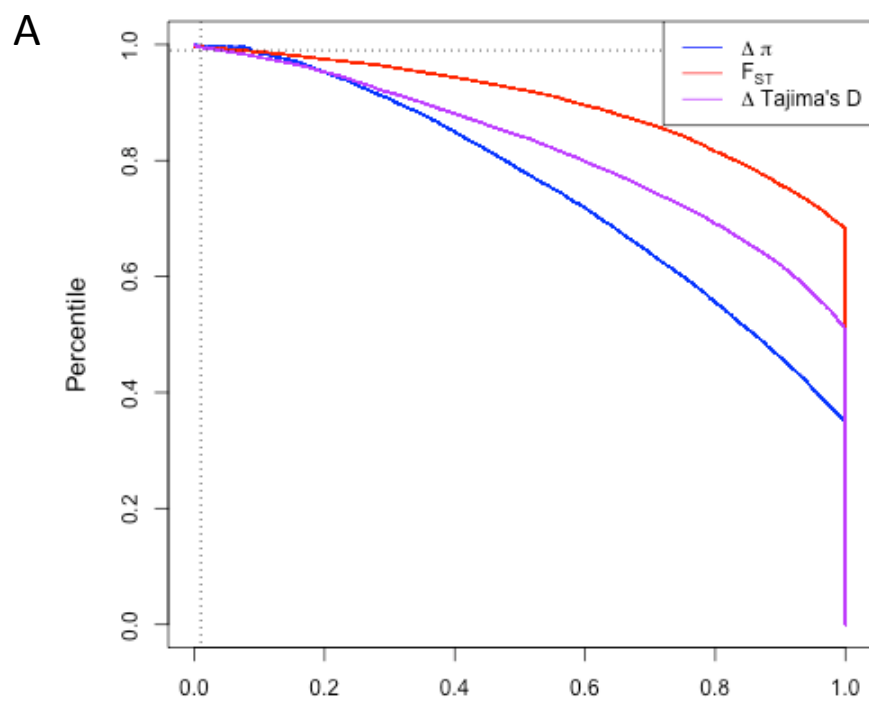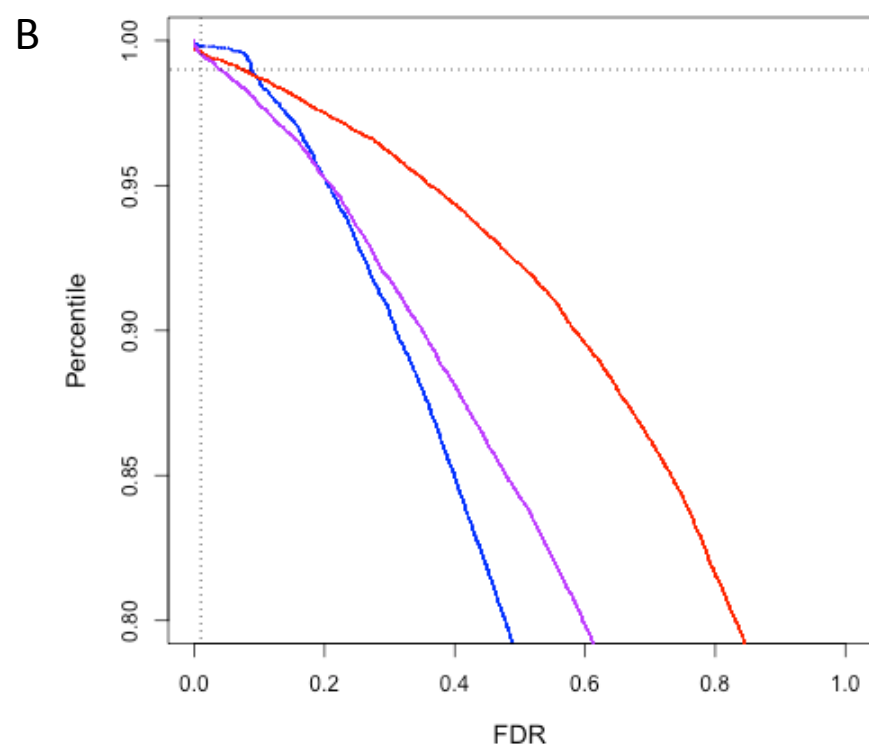

Supplement: S4 Fig — (A) Entire range of empirical percentile and (B) Focus on the top 20% of the empirical distribution. Horizontal and vertical dotted lines indicate the 99th percentile and 1% FDR, respectively. (PDF) [file pgen.1005851.s006.pdf]

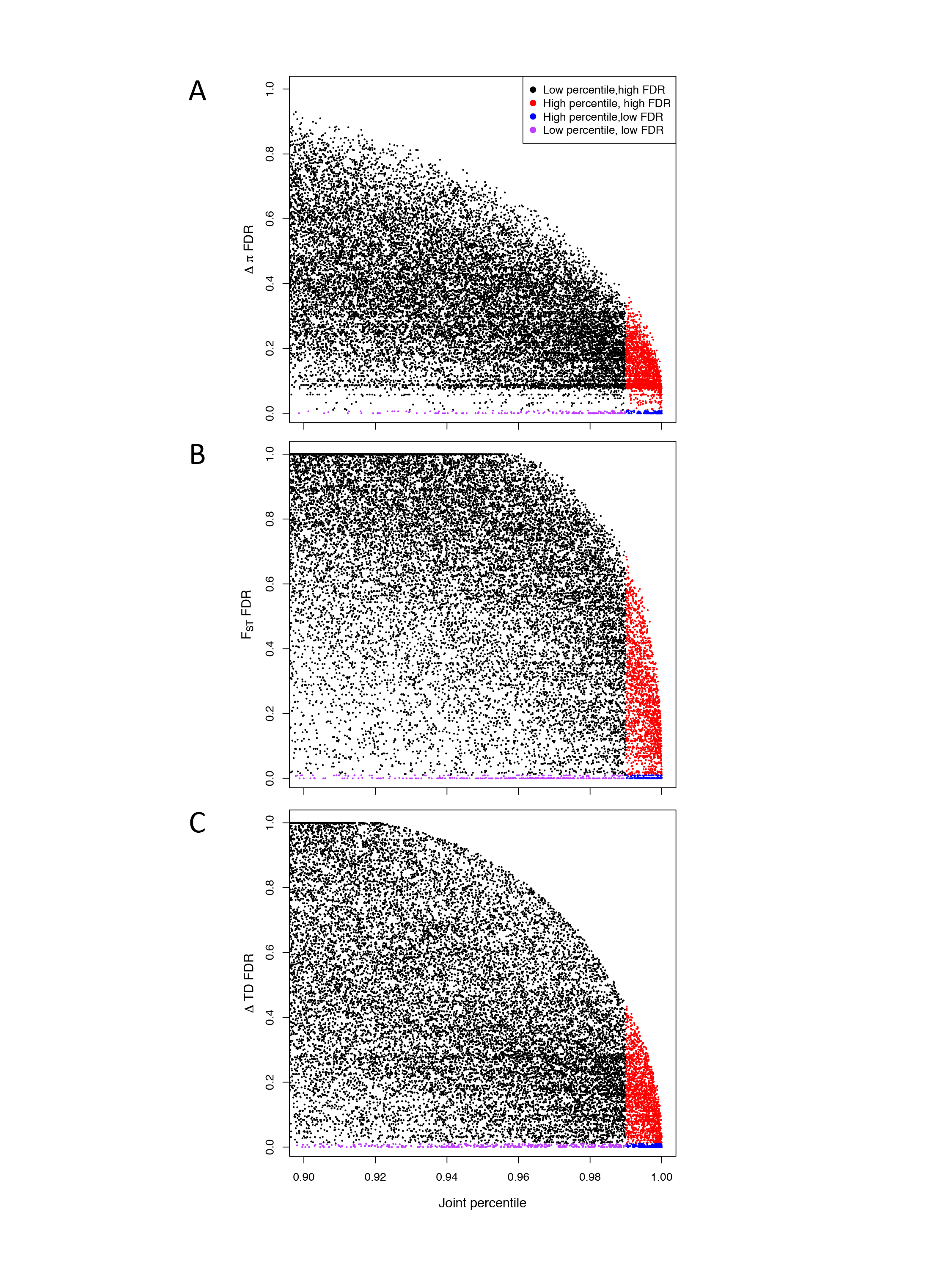

Supplement: S5 Fig — FDR of individual statistics vs. the joint percentile statistic for 100kb windows, used to identify outlier windows in the empirical outlier (non-FDR) approach, for (A) Δπ, (B) FST, and (C) Δ Tajima’s D. (PNG) [file pgen.1005851.s007.png]

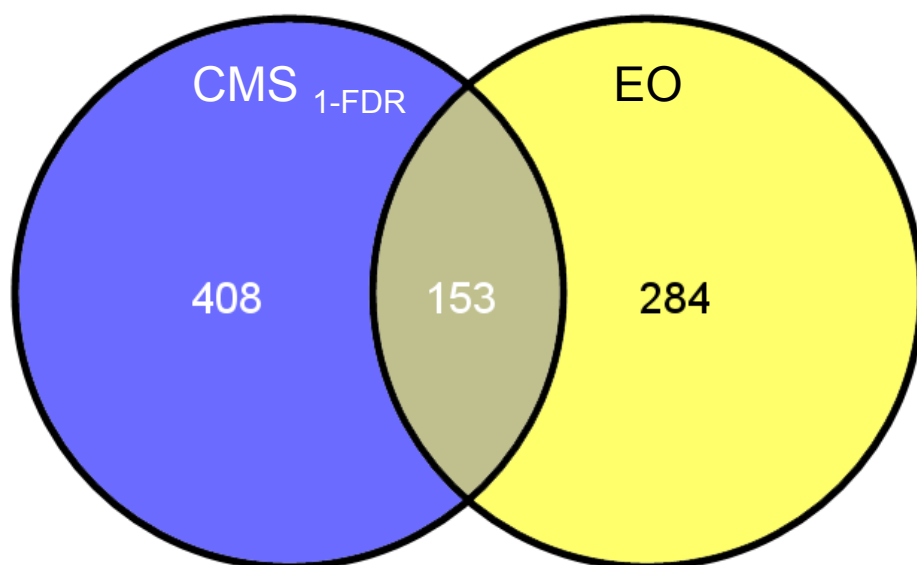

Supplement: S6 Fig — Genes unique to empirical methods relative to FDR methods are those falling within windows with a high false discovery rate (and thus are likely to be enriched with false positives) (PDF) [file pgen.1005851.s008.pdf]

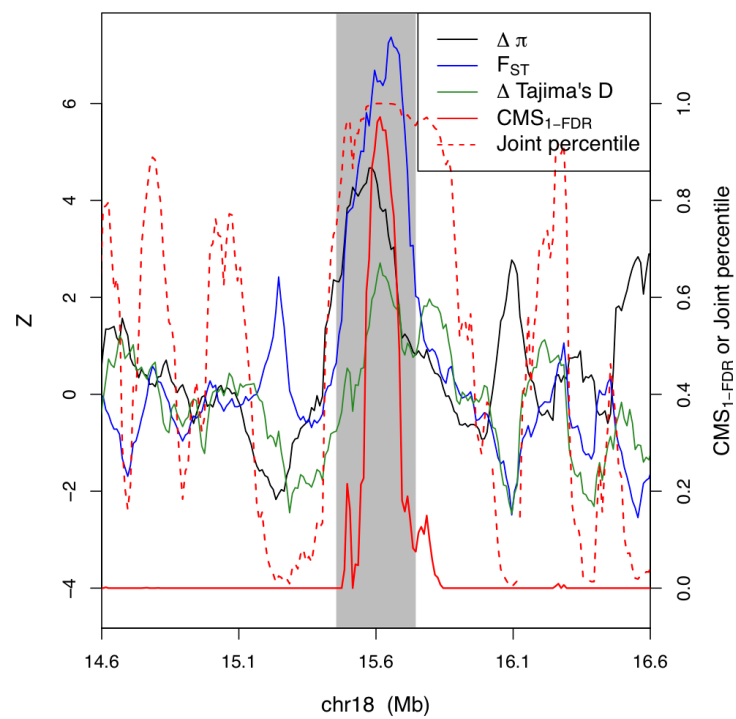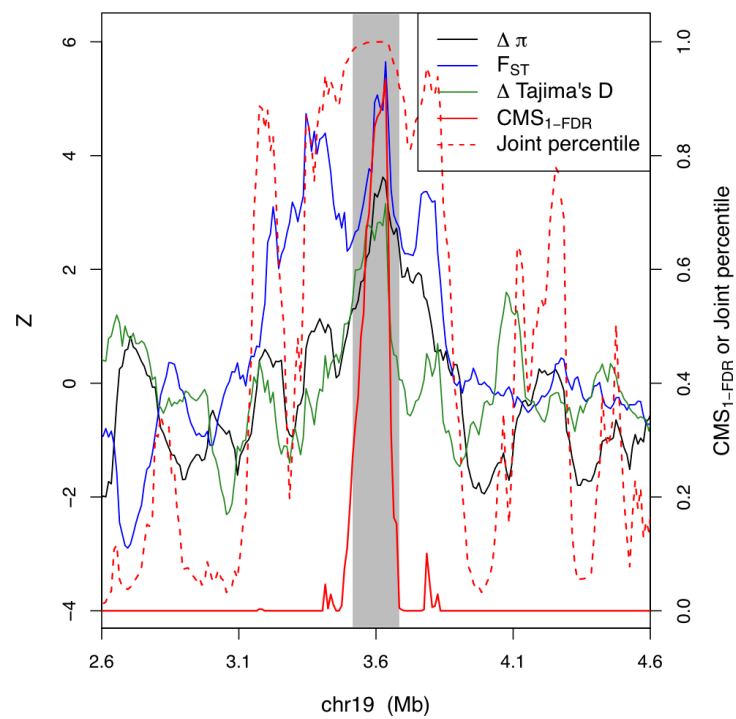

Supplement: S7 Fig — (PDF) [file pgen.1005851.s009.pdf]

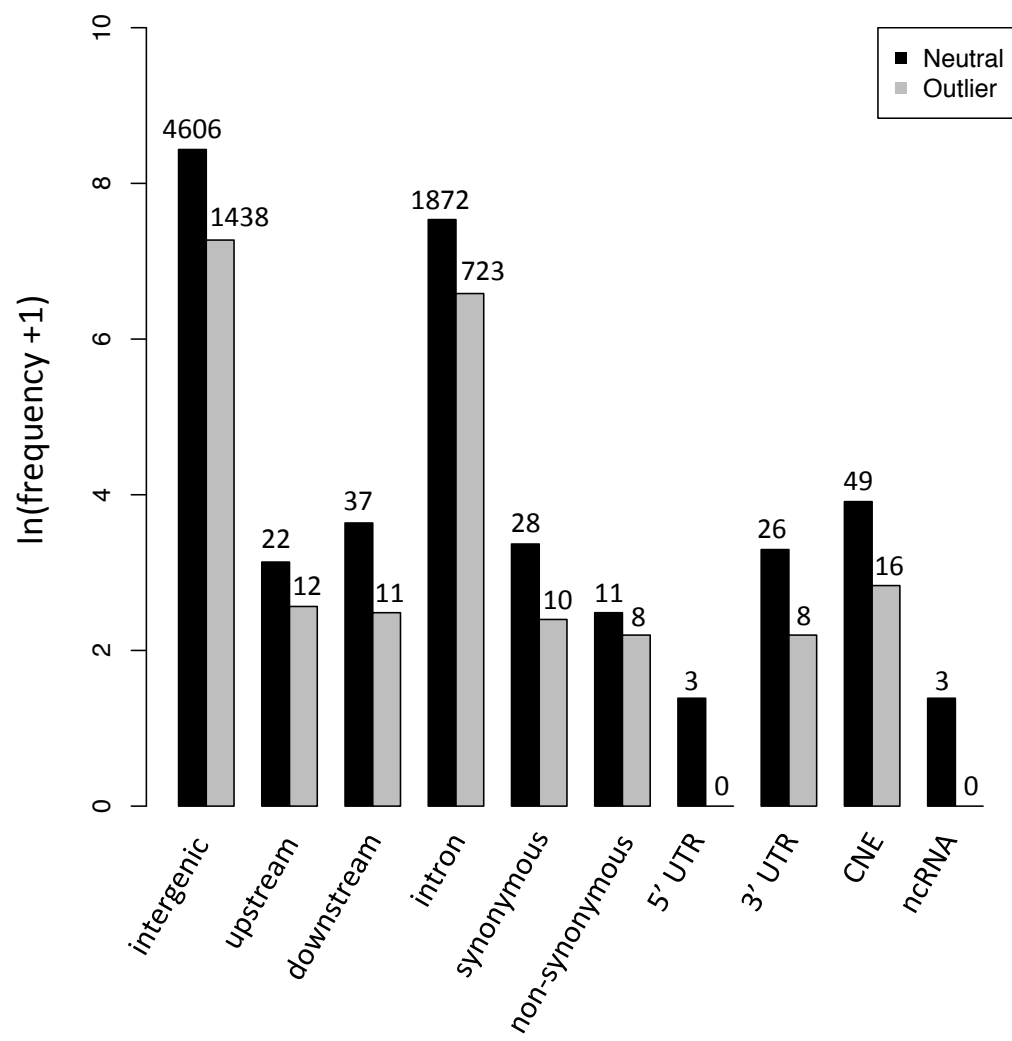

Supplement: S9 Fig — Numbers above bars indicate counts of fixed sites. (PDF) [file pgen.1005851.s011.pdf]
