# Supplementary material for: Demographically-Based Evaluation of Genomic Regions under Selection in Domestic Dogs
Source: PLoS Genet. 2016 Mar 4;12(3):e1005851. doi: 10.1371/journal.pgen.1005851 (PMC4778760; doi:10.1371/journal.pgen.1005851)
Supplement: S8 Fig — Columns within “This study” are based on the sequencing data generated here, while those under CanMap are computed from a ~48k SNP data set for a large set of wolves and ancient/basal dog breeds. Heat map colors reflect upper percentiles of the calculated metrics, with warmer colors indicating higher percentiles. Overlaps with previous studies: 1, vonHoldt et al. 2010 [35]; 2, Vaysse et al. (2011) [25]; 3, Boyko et al. (2010) [23]; and Axelsson et al.(2013), [27], with numbers indicating the joint percentile, FST, FST, and region id, respectively for each study. (PDF) [file pgen.1005851.s010.pdf]

Region	This Study Δπ	This Study Fst	This Study ΔTD	This Study CMS_{1-FDR}	CanMap Data Δπ	CanMap Data Fst	CanMap Data Joint	Other Selection Studies 1	Other Selection Studies 2	Other Selection Studies 3	Other Selection Studies 4	Genes
chr18:15.49–15.78	4.03	0.63	1.74	0.97	0.76	0.7	99.25	94.8				LHFPL3
chr18:0.57–1.02	4.36	0.67	1.62	0.97							28	
chr19:3.55–3.72	3.16	0.51	2.09	0.93	0.58	0.83	99.22	93.1				CCRN4L
chr31:1.95–2.14	2.9	0.65	2.17	0.92	0.54	0.4	94.22					CADM2
chr12:59.02–59.17	3.93	0.64	1.32	0.92	−1.06	0.01	0.57					RPL3;ACA64
chr10:4.04–4.22	2.42	0.53	2.21	0.9	0.3	0.61	96.02				18	SNORA31
chr11:37.55–37.76	3.18	0.69	1.62	0.89	0.03	0.47	79.33					SH3GL2
chr24:4.37–4.59	2.17	0.59	1.91	0.89	0.12	0.12	48.36					DTD1
chr24:23.25–23.48	8.91	0.69	1.4	0.89					0.7	0.64		ASIP;AHCY;unknown
chr02:21.9–22.16	1.87	0.93	2.14	0.88	0.18	0.19	68.71					FAM107B
chr32:0.64–0.78	4.09	0.43	2.66	0.88								ART3;NUP54;unknown;SCARB2
chr13:46.85–47.02	3.54	0.42	2	0.87	0.19	0.54	92.4	92.5				
chr13:53.99–54.13	3.71	0.43	1.65	0.87	0.05	0.3	75.43					unknown
chr01:2.93–3.19	2.75	0.75	2.13	0.86	−0.04	0.13	40.36				1	MBP;ZNF236
chr01:28.36–28.52	2.83	0.6	1.52	0.85	0.1	0.51	87.23	93.3				PDE7B
chr02:46.35–46.53	3.19	0.43	2.4	0.85	−1.39	0.34	3.67					
chr06:28.39–28.49	2.1	0.45	1.79	0.83	0.24	0.52	93.8					unknown;PDXDC1;NTAN1;RRN3
chr04:57.36–57.48	2.49	0.45	1.87	0.82	0.16	0.62	91.71					GLRA1
chr37:16.14–16.27	1.9	0.46	2.5	0.82	0.47	0.67	98.18	91.5				PLEKHM3
chr04:60.02–60.27	3.71	0.6	1.01	0.81	−0.14	0.52	54.44					
chr15:9.83–9.93	3.48	0.47	1.12	0.81	−0.36	0.4	31.01					EPS15;TTC39A
chr08:71.21–71.31	3.11	0.4	1.79	0.8								MARK3;U2;CKB;TRMT61A;BAG5;APOPT1
chr04:61.87–62.1	4.16	0.43	1.89	0.8	0.04	0.21	64.58					unknown
chr16:39.84–39.99	3.44	0.39	2.15	0.8	−0.11	0.07	19.51					
chr04:4.17–4.34	2.54	0.42	2.04	0.8	−1.29	0.06	1.34					LYST;unknown;GNG4
chr17:5.62–5.74	1.79	0.55	1.78	0.78	0.74	0.37	93.72					
chr11:48.48–48.71	5.07	0.4	1.58	0.78								
chr10:3.72–3.98	5.07	0.39	1.44	0.78							18	
chr36:0.34–0.52	1.68	0.67	1.63	0.77								MINOS1
chr02:53.12–53.38	2.24	0.41	2.45	0.77	0.27	0.41	91.98					
chr26:8.68–8.79	2.91	0.38	1.86	0.75	−0.05	0.1	29.52					CUX2
chr01:46.69–46.83	4.02	0.4	1.17	0.75	−0.15	0.11	26.5				2	ARID1B
chr18:24.04–24.16	2.41	0.39	1.89	0.75				90.3				
chr28:8.2–8.3	1.55	0.42	1.81	0.74	0.31	0.56	95.59	97.4				PLCE1
chr14:50.91–51.34	2.49	0.43	2.66	0.74	−0.7	0.48	15.13					LRRN3
chr34:14.47–14.61	2.24	0.55	0.97	0.73								unknown
chr04:40.19–40.59	0.98	0.61	2.11	0.73	0.06	0.05	19.88					C5orf50
chr26:2.42–2.61	3.4	0.67	0.96	0.72	−0.01	0.65	73.88					TMEM132D
chr34:26.31–26.51	1.4	0.78	2.34	0.72	−0.09	0.24	51.37					IFT80;SMC4;cfa–mir–15b;cfa–mir–16–2;TRIM59
chr11:46.55–46.92	4.23	0.64	1.14	0.7								cfa–mir–876;unknown
chr16:4.83–4.93	2.24	0.56	0.86	0.69	0.81	0.76	99.56					
chr11:9.72–9.88	5.26	0.41	0.9	0.68	0.08	0.13	49.56					
chr04:40.74–41.09	1.2	0.87	1.26	0.68	−0.96	0.07	2.91				10	RANBP17
chr06:25.29–25.4	8.82	0.44	0.94	0.67								GPR139;GPRC5B
chr32:0.53–0.63	1.31	0.39	1.8	0.67								SDAD1;unknown;CXCL10
chr02:43.98–44.08	3.51	0.35	1.37	0.67	−0.08	0.02	7.12					MAP3K1;SETD9;MIER3
chr31:3.09–3.19	2.29	0.35	1.77	0.67				93.9				
chr24:23.83–23.93	3.38	0.45	0.73	0.66	0.38	0.27	84.69					NCOA6;GGT7;ACSS2
chr18:1.46–1.71	3.34	0.55	1.83	0.66								C7orf72;IKZF1
chr37:4.38–4.57	4.29	0.42	0.77	0.63								
chr25:6.73–6.86	4.14	0.37	0.97	0.63								unknown
chr18:24.26–24.38	3.64	0.34	1.21	0.62	0.29	0.55	95.28	90.3				SEMA3D
chr01:79.87–80.05	3.11	0.58	0.63	0.61							4	unknown
chr21:7.19–7.34	2.2	0.33	2.12	0.6	0.91	0.93	99.85					unknown;CCDC67
chr18:0.45–0.56	3.62	0.4	0.88	0.59							28	
chr04:41.7–41.83	1.47	0.58	0.73	0.59	−0.57	0.18	14.6					FOXI1;DOCK2
chr37:6.56–6.66	2.8	0.55	0.59	0.59	0.28	0.15	60.86					unknown
chr13:48.69–48.82	1.59	0.34	2.05	0.59	0.22	0.3	85.08					SRP72;ARL9;THEGL;HOPX
chr10:6.39–6.5	2.92	0.56	0.53	0.57	−0.11	0.14	35.56					
chr06:33.34–33.56	4.08	0.65	1.02	0.56	−0.11	0.29	52.43					ABAT;METTL22;unknown;TMEM114
chr11:37.8–37.94	3.48	0.5	0.59	0.54							19	
chr01:21.15–21.26	2.2	0.58	0.49	0.53	0.33	0.5	95.13					STARD6;POLI;MBD2
chr16:2.08–2.24	1.39	0.56	0.61	0.52	−0.01	0	0.5					unknown
chr12:58.03–58.13	3.91	0.34	0.74	0.5	0.45	0.19	72.03					MCHR2
chr24:20.91–21.02	1	0.32	1.96	0.5	0.15	0.15	57					DEFB122;CBD118;DEFB119;CBD121;CBD122;DEFB3L;CBD123;DEFB124;CBD124;REM1;[illegible]
chr01:50.78–50.92	1.23	0.31	1.88	0.48	0.42	0.45	95	95.8				unknown
chr37:12.31–12.42	1.58	0.3	1.94	0.48	0.34	0.38	92.04					RAPH1;unknown;U2
chr16:18.74–18.88	1.64	0.31	1.9	0.48	0.37	0.64	97.09					
